# Kernel mean matching enhances risk estimation under spatial distribution shifts

**DOI:** 10.1038/s41598-026-36740-7

**Published:** 2026-02-02

**Authors:** Egor Serov, Diana Koldasbayeva, Alexey Zaytsev

**Affiliations:** 1 Skolkovo Institute of Science and Technology, Moscow, Russia; 2 Beijing Institute of Mathematical Sciences and Applications, Beijing, China

**Keywords:** Kernel mean matching, Spatial risk estimation, Spatial modeling, Importance reweighting, Distribution shift robustness, Computational biology and bioinformatics, Ecology, Ecology, Mathematics and computing

## Abstract

Accurate risk estimation under distribution shifts is critical for deploying machine learning models in real-world spatial applications, from ecological forecasting to medical image analysis. Conventional methods such as No Weighting (NW) and Importance Weighting (IW) fail in spatially structured data due to two challenges: (1) density ratio estimation in high-dimensional clustered distributions and (2) non-stationarity from environmental gradients or sampling biases. Classifier-based approaches offer partial improvements but often yield miscalibrated risk estimates by prioritizing discriminative accuracy over distribution alignment. We conduct a systematic evaluation of four risk estimation methods —NW, IW, Kernel Mean Matching (KMM), and classifier-based reweighting—across synthetic benchmarks (with controlled spatial clustering) and real-world datasets (species distributions and immune cell layouts). Results show that KMM achieves superior robustness, reducing Mean Absolute Percentage Error (MAPE) by 12.3–86.5% compared to alternatives in high-dimensional settings. This advantage stems from KMM’s direct minimization of distributional divergence via kernel embeddings, bypassing error-prone density ratio estimation. Our findings demonstrate that KMM is a principled solution for spatial risk estimation, particularly when source and target distributions exhibit complex clustering or sampling artifacts. Its consistency across ecological and biomedical domains suggests broad applicability for reliable model deployment in spatially heterogeneous environments.

## Introduction

The risk of a model is the expected error of a data-based model on unseen data. Reliable risk estimation justifies or invalidates the use of a particular model, allowing a practitioner to assess its utility. Under independent and identically distributed (i.i.d.) assumptions, cross-validation and hold-out testing provide theoretically sound estimates. However, these methods can fail dramatically under distribution shift between training and test data, particularly in spatial settings where data exhibit complex dependencies. One example is covariate shift, where the distribution of a model input changes when switching from source (training) to target (test) data. Under the covariate shift, traditional estimators systematically underrate the true error^1^. This issue is especially problematic in scientific and environmental applications, where overly optimistic error estimates can lead to incorrect conclusions.

For example, the simplest estimator, No Weighting (NW), computes the empirical error directly from available target samples. It implicitly assumes that the source and target distributions are similar. Under covariate shift, NW becomes biased^2^. A more flexible family of estimators trains a probabilistic classifier to distinguish source from target data and converts its outputs into density-ratio weights^3,4^. Although often more accurate than NW, classifier-based weighting inherits instability from imperfect class separation and remains sensitive to clustered or non-overlapping samples. The classical importance weighting (IW) estimator rescales source samples using the density ratio between target and source distributions. While IW is theoretically unbiased, it suffers from extreme weight variance in high dimensions or under sparse sampling^5^.

These challenges become more severe when data exhibit spatial structure. Spatial datasets are affected by non-stationarity, spatial autocorrelation, clustered sampling, and environmental gradients that induce strong distribution shifts. For example, mismatches between observed and modeled sea surface temperature trends indicate that standard climate models fail to capture important components of real-world climate dynamics^6^. Similarly, climate-driven shifts toward water-limited regimes are transforming terrestrial ecosystems, altering vegetation dynamics and ecosystem services^7^. Spatial biases also manifest in species-distribution data^8^, pollution monitoring^9^, and numerous biomedical settings.

In biomedical research, spatial artifacts strongly influence analyses^10^. A common pitfall occurs when adjacent normal tissue is used as a control in cancer studies: despite being anatomically normal, it is biologically altered by tumour-proximal effects, leading to biased differential-expression estimates^11^. Kernel Maximum Mean Discrepancy (MMD) has been used to quantify these distributional differences and has shown improved sensitivity in identifying perturbed genes and pathways^11,12^. Spatial clustering is also intrinsic to tumour–immune microenvironments^13^, further complicating risk estimation.

Several methodological frameworks address spatial dependence. Spatial and spatio-temporal cross-validation^14^ yields more realistic predictive performance estimates than random splitting but does not correct for covariate shift: it evaluates generalization under structured partitioning rather than reweighting samples to match the target distribution. Diagnostic tools such as the Area of Applicability (AOA)^15^ quantify how dissimilar a target location is from the training domain but do not estimate the model’s error under shift.

Domain adaptation methods^16,17^ aim to improve predictive accuracy by aligning source and target distributions, yet their goal is optimization—not evaluation. In many scientific pipelines, the model is fixed, and the task is solely to estimate its error under distribution shift. Thus, domain adaptation and risk estimation solve fundamentally different problems.

Across the literature, a common limitation emerges: existing tools either assume i.i.d. data, diagnose shift without estimating error, or modify the model rather than evaluating it. Crucially, none provide a stable, unbiased risk estimator under *spatial covariate shift*, where clustered sampling, autocorrelation, and non-stationarity break classical density estimation and destabilize classifier-based ratio estimation.

To address this gap, we formulate spatial risk estimation as a sample–reweighting problem and systematically evaluate reweighting strategies under spatial covariate shift. We show that Kernel Mean Matching (KMM)^18^, originally proposed for covariate shift correction, yields stable and accurate risk estimates for spatially structured data. To quantify spatial structure, we incorporate the Local Correlation Function (LCF)^19^, a bounded, scale-invariant measure of spatial clustering, which provides an interpretable criterion for when reweighting is necessary.

Through experiments on synthetic Gaussian-mixture landscapes, Nordic plant-species occurrences, and tumour–immune spatial layouts, we demonstrate that KMM reduces Mean Absolute Percentage Error (MAPE) by up to 50% compared with IW while avoiding its weight-explosion pathology.

**Fig. 1:**
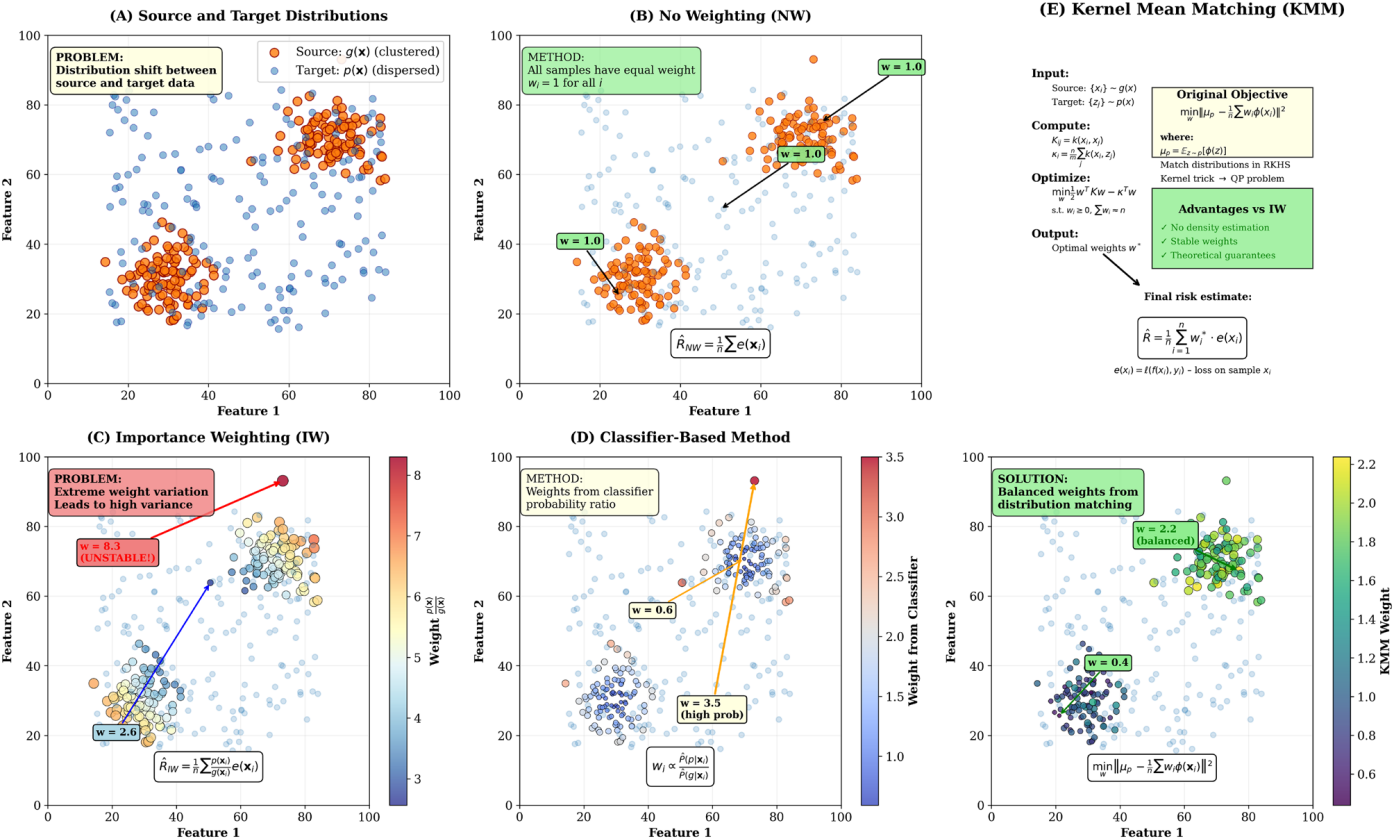
Visual comparison of risk estimation methods under distribution shift: (**A**) Problem formulation showing source (clustered) and target (dispersed) distributions; (**B**) No Weighting (NW) approach with uniform sample weights; (**C**) Importance Weighting (IW) method suffering from high variance; (**D**) Classifier-based probability ratio weighting; (**E**) Kernel Mean Matching (KMM) approach: upper section shows the optimization framework that matches source and target distributions, while lower section demonstrates the resulting balanced weight distribution that avoids extreme values while correcting for distribution shift.

As illustrated in Fig.  1, our comparison highlights the trade-offs among NW, IW, classifier-based weighting, and KMM, showing that direct distribution matching offers a robust solution for risk estimation under spatial covariate shift.

- We formulate spatial risk estimation as a reweighting problem for sample errors, extending classical importance-sampling theory to spatially structured settings in which source and target distributions differ. Our pipeline integrates the LCF score^19^ as an interpretable, scale-invariant measure of spatial clustering that indicates when reweighting is likely to be beneficial.
- We conduct a systematic empirical comparison of NW, IW, KMM, and a classifier-based estimator across synthetic and real-world spatial datasets, covering classification tasks and assessing risk estimation via regression-style loss metrics. Our evaluation spans (i) controlled Gaussian-mixture landscapes and other synthetic scenarios, (ii) Nordic plant-species occurrences, and (iii) tumour–immune cell layouts. Our experiments reveal systematic biases in risk estimation induced by shifts in spatial distribution and support LCF as a practical diagnostic of shift magnitude.
- Across all datasets considered, KMM remains a robust choice for spatial risk estimation under distribution shift, particularly when shifts are complex or labelled samples are limited. Specifically, the usage of KMM reweighting reduces the risk estimation error (MAPE) by up to 50% relative to IW while avoiding the weight-explosion pathologies of KDE-based density-ratio estimation for severe shifts.

## Methods

### Risk estimation task

We begin with a ground truth function $f({\textbf{x}})$ and a model that estimates this function, denoted as ${\hat{f}}({\textbf{x}})$. These functions are defined as maps from some region ${\mathscr {X}} \subseteq {\mathbb {R}}^d$ to ${\mathbb {R}}$ for a regression problem. In this study, we explicitly assume that ${\mathscr {X}}$ resides in a $d$-dimensional Euclidean space, utilizing the standard Euclidean metric, though the framework implies potential generalizability to other metric spaces. Next, we have labeled samples from the source distribution $g({\textbf{x}})$, on which we can evaluate our model using some error function $e({\textbf{x}}) = e(f({\textbf{x}}), {\hat{f}}({\textbf{x}}))$, defined in ${\mathscr {X}}$.

We are interested in the performance of our model ${\hat{f}}({\textbf{x}})$ on unlabeled points from another target distribution $p({\textbf{x}})$. The function $p({\textbf{x}})$ is similarly defined on the same region. The key challenge is that $p({\textbf{x}})$ and $g({\textbf{x}})$ are different: we deal with a distribution shift that leads to bias. Moreover, we lack the exact $p({\textbf{x}})$ and $g({\textbf{x}})$ and have access only to samples from them $D_p, D_g$: $D_p = \{({\textbf{x}}_i, f({\textbf{x}}_i))\}_{i = 1}^{n_p}$, ${\textbf{x}}_i \sim p({\textbf{x}})$, $D_g = \{({\textbf{x}}_i, f({\textbf{x}}_i))\}_{i = 1}^{n_g}$, ${\textbf{x}}_i \sim g({\textbf{x}})$ with $n_p = |D_p|, n_g = |D_g|$. The source and target data are split into training sets, $D_g^{\textrm{train}}$ and $D_p^{\textrm{train}}$, and test sets, $D_g^{\textrm{test}}$ and $D_p^{\textrm{test}}$, with respective sample sizes $n_g^{\textrm{train}}$, $n_p^{\textrm{train}}$, and $n_g^{\textrm{test}}$, $n_p^{\textrm{test}}$. Formally, sample sizes can differ, and specific points are also distinct by construction, as they are generated from different distributions.

Formally, we are interested in estimating the risk:

1 $$\begin{aligned} R(e, p) = \int _{{\mathscr {X}}} e({\textbf{x}}) p({\textbf{x}}) d{\textbf{x}}. \end{aligned}$$

In practice, the exact forms of the distributions $p({\textbf{x}})$ and $g({\textbf{x}})$ are unknown, and ground truth labels $f({\textbf{x}})$ for the target distribution are unavailable. Therefore, the estimation of the risk $R(e, p)$ must rely only on the following components:

- The model to be evaluated: ${\hat{f}}({\textbf{x}})$.
- A set of labeled samples from the source distribution: $D_g$.
- A set of unlabeled samples from the target distribution: $\{{\textbf{x}}_j\}_{j = 1}^{n_p}$, where ${\textbf{x}}_j \sim p({\textbf{x}})$.

Below, we provide general types of solutions for risk estimation tasks. To address the challenge of estimating risk under a distribution shift, we explore a spectrum of methods. Our selection is motivated by the need to establish a clear performance hierarchy, starting from a naive baseline and progressing to more sophisticated, theoretically-grounded techniques for bias correction. We begin with the simplest approach, which ignores the distribution shift, to quantify the magnitude of the problem. Subsequently, we examine methods that explicitly attempt to correct the bias introduced by the shift. These corrective methods fall into two main categories: those that rely on estimating the density ratio either directly or indirectly, and those that match the distributions in a feature space without explicit density estimation. This progression enables a comprehensive evaluation of various strategies for addressing the covariate shift problem. The final performance of each method will be validated on the test data partitions, where risk estimates are computed using $D_g^{\textrm{test}}$ and benchmarked against the ground truth risk calculated on $D_p^{\textrm{test}}$.

#### No weighting (NW)

The most straightforward approach would be to estimate the risk using samples from the target distribution $p({\textbf{x}})$:

2 $$\begin{aligned} {\hat{R}}_{GT}(e, p) = \frac{1}{n_p^{\textrm{test}}} \sum _{i=1}^{n_p^{\textrm{test}}} e({\textbf{x}}_i), \quad {\textbf{x}}_i \sim p({\textbf{x}}). \end{aligned}$$

To compute this value, we would hypothetically use the test sample $D_p$ with its corresponding labels. This is an unbiased Monte Carlo estimate of the true risk *R*(*e*, *p*), and its standard error decreases at a rate of $O(1/\sqrt{n_p^{\textrm{test}}})$. Consequently, for a sufficiently large sample size *n*, ${\hat{R}}_{GT}$ provides a highly accurate and reliable benchmark against which alternative risk estimation methods can be validated. This is a typical method for estimating the risk of samples from a known distribution. However, as mentioned earlier, in practice, we do not have labeled samples from the target distribution $p({\textbf{x}})$, but we do have labeled samples from another source distribution $g({\textbf{x}})$.

The first idea is to estimate the risk similarly, but using samples from the source distribution. Thus, we have the NW method:

3 $$\begin{aligned} {\hat{R}}_{NW}(e, g) = \frac{1}{n_g^{\textrm{test}}} \sum _{i=1}^{n_g^{\textrm{test}}} e({\textbf{x}}_i), \quad {\textbf{x}}_i \sim g({\textbf{x}}). \end{aligned}$$

In the presence of a distribution shift, this method is obviously biased, and we should strive for an unbiased approach if our goal is to estimate the target risk accurately.

#### Importance weighting (IW)

To correct for the bias introduced by using samples from $g({\textbf{x}})$ instead of $p({\textbf{x}})$, we can apply IW. Using this technique, we reweight the errors across samples according to the density ratio $\frac{p({\textbf{x}})}{g({\textbf{x}})}$. This is an intuitive way to achieve an unbiased risk estimate. Therefore, the risk can be expressed as follows:

4 $$\begin{aligned} R(e, p) = \int _{\mathscr {X}} e({\textbf{x}}) p({\textbf{x}}) \, d{\textbf{x}}= \int _{\mathscr {X}} e({\textbf{x}}) p({\textbf{x}}) \frac{g({\textbf{x}})}{g({\textbf{x}})} \, d{\textbf{x}}= \int _{\mathscr {X}} e({\textbf{x}}) \frac{p({\textbf{x}})}{g({\textbf{x}})} g({\textbf{x}}) \, d{\textbf{x}}. \end{aligned}$$

Thus, instead of integrating the error $e({\textbf{x}})$ over the target distribution, we can incorporate the reweighted error $\frac{p({\textbf{x}})}{g({\textbf{x}})} e({\textbf{x}})$ over the source distribution $g({\textbf{x}})$, resulting in a new unbiased risk estimation. We refer to this as IW:

5 $$\begin{aligned} R_{IW}(e, p, g) = \frac{1}{n_g^{\textrm{test}}} \sum _{i=1}^{n_g^{\textrm{test}}} \frac{p({\textbf{x}}_i)}{g({\textbf{x}}_i)} e({\textbf{x}}_i), \quad {\textbf{x}}_i \sim g({\textbf{x}}). \end{aligned}$$

In practice, the true densities $p({\textbf{x}})$ and $g({\textbf{x}})$ are unknown. We should estimate $g({\textbf{x}})$ and $p({\textbf{x}})$ from the data. For example, using KDE and samples $D_p^{\textrm{train}}$ and $D_g^{\textrm{train}}$, we get ${\hat{g}}({\textbf{x}})$ and ${\hat{p}}({\textbf{x}})$.

An example of the kernel density estimator for a multivariate density $g(\mathbf {{\textbf{x}}})$, where $\mathbf {{\textbf{x}}} \in {\mathbb {R}}^d$, is given by:

6 $$\begin{aligned} {\hat{g}}(\mathbf {{\textbf{x}}}) = \frac{1}{n_g^{\textrm{train}}} \sum _{i=1}^{n_g^{\textrm{train}}} |H|^{-1/2} K\left( H^{-1/2}(\mathbf {{\textbf{x}}} - \mathbf {{\textbf{x}}}_i)\right) , \end{aligned}$$

where $K(\cdot )$ is the multivariate kernel function, chosen as a multivariate Gaussian kernel:

7 $$\begin{aligned} K({\textbf{u}}) = \frac{1}{(2\pi )^{d/2}} \exp \left( -\frac{1}{2} {\textbf{u}}^\top {\textbf{u}} \right) , \end{aligned}$$

and $H \in {\mathbb {R}}^{d \times d}$ is the symmetric positive-definite bandwidth matrix that controls the smoothness of the estimate.

We select the bandwidth matrix $H$ using Scott’s rule^20^ :

8 $$\begin{aligned} H = \Sigma \left( n_g^{\textrm{train}}\right) ^{-\frac{2}{d+4}} \end{aligned}$$

where $\Sigma$ is the sample covariance matrix of the data. We also considered using a fixed bandwidth. However, a constant value does not adapt to the specific properties of the data, such as its scale or sample size. Scott’s rule is a data-driven method that uses the sample covariance and size to determine the bandwidth. This adaptability generally leads to more reliable density estimates.

Using this method, we estimate the density functions $g({\textbf{x}})$ and $p(x)$ from the data and apply them in the IW:

9 $$\begin{aligned} {\hat{R}}_{IW}(e,{\hat{p}},{\hat{g}}) = \frac{1}{n_g^{\textrm{test}}} \sum _{i=1}^{n_g^{\textrm{test}}} e({\textbf{x}}_i) \frac{{\hat{p}}({\textbf{x}}_i)}{{\hat{g}}({\textbf{x}}_i)}, \quad {\textbf{x}}_i \sim g({\textbf{x}}). \end{aligned}$$

Challenges with High Weights for IW One key problem with IW is that when the density ratio $p({\textbf{x}})/g({\textbf{x}})$ is large, specific samples receive excessively high weights, leading to high variance of the estimate. This occurs especially in regions where $g({\textbf{x}})$ is significantly smaller than $p({\textbf{x}})$, resulting in instability in the risk estimate and poor performance of the IW method compared to other methods, regardless of bandwidth selection. We applied LCF function analysis to the spatial features of datasets from our domain. The results reveal a stark contrast in their spatial structure. The source distribution exhibits a high degree of clustering, with data points concentrated in specific areas. In contrast, the target distribution is significantly less clustered, approaching a random spatial pattern. This structural mismatch means that the density ratio can become extremely large, particularly in regions where the source distribution is sparse but the target is not. This leads to high variance in the importance weights, destabilizing the IW estimator.

#### Classifier method

This approach^3,4^ utilizes a probabilistic classifier to directly estimate the density ratio $p({\textbf{x}})/g({\textbf{x}})$ instead of independent estimates of $p({\textbf{x}})$ and $g({\textbf{x}})$. These ratio estimates would serve as importance weights for risk estimation. The fundamental idea is to train a classifier to discriminate between samples originating from the source distribution $g({\textbf{x}})$ and those from the target distribution $p({\textbf{x}})$.

To begin, a dedicated training dataset is constructed for the auxiliary classifier using our training partitions. This dataset is formed by taking the features from the source training set $D_g^{\textrm{train}}$ and the features from the target training set $D_p^{\textrm{train}}$. These combined samples are then assigned new binary labels: samples originating from $D_g^{\textrm{train}}$ are labeled as class 0, and samples from $D_p^{\textrm{train}}$ are labeled as class 1.

The number of samples in this new training set from the source and target distributions are $n_g^{\textrm{train}}$ and $n_p^{\textrm{train}}$, respectively. The empirical prior probabilities for an instance belonging to the source class or the target class are then:

$$\begin{aligned} {\hat{\pi }}_{\mathrm {g-class}}&= \frac{n_g^{\textrm{train}}}{n_g^{\textrm{train}} + n_p^{\textrm{train}}}, \\ {\hat{\pi }}_{\mathrm {p-class}}&= \frac{n_p^{\textrm{train}}}{n_g^{\textrm{train}} + n_p^{\textrm{train}}}. \end{aligned}$$

A probabilistic classifier, such as Gradient Boosting, is trained on this aggregated and labeled dataset. Using the empirical priors ${\hat{\pi }}_{\mathrm {g-class}}$ and ${\hat{\pi }}_{\mathrm {p-class}}$, the classifier learns to model the posterior probability via Bayes’ rule. Once trained, for any given input instance ${\textbf{x}}$, this classifier can provide an estimate of the probability that ${\textbf{x}}$ belongs to the class associated with the target distribution $p({\textbf{x}})$, denoted as ${\hat{P}}(p|{\textbf{x}})$, and consequently, the probability it belongs to the class associated with the source distribution $g({\textbf{x}})$, ${\hat{P}}(g|{\textbf{x}}) = 1 - {\hat{P}}(p|{\textbf{x}})$.

The crucial insight is that the density ratio $p({\textbf{x}})/g({\textbf{x}})$ can be estimated using the outputs of this classifier. For a sample ${\textbf{x}}_i$ drawn from the source distribution $g({\textbf{x}})$, the estimated importance weight $w_i$, which approximates the true density ratio $p({\textbf{x}}_i)/g({\textbf{x}}_i)$, is given by:

10 $$\begin{aligned} w_i = \frac{{\hat{\pi }}_{\text {g-class}}}{{\hat{\pi }}_{\text {p-class}}} \frac{{\hat{P}}(p|{\textbf{x}}_i)}{{\hat{P}}(g|{\textbf{x}}_i)}. \end{aligned}$$

Substituting the empirical priors and classifier probabilities, this expression becomes:

11 $$\begin{aligned} w_i = \frac{n_g^{\textrm{train}} / (n_g^{\textrm{train}} + n_p^{\textrm{train}})}{n_p^{\textrm{train}} / (n_g^{\textrm{train}} + n_p^{\textrm{train}})} \left( \frac{1-{\hat{P}}(g|{\textbf{x}}_i)}{{\hat{P}}(g|{\textbf{x}}_i)}\right) = \frac{n_g^{\textrm{train}}}{n_p^{\textrm{train}}} \left( \frac{1}{{\hat{P}}(g|{\textbf{x}}_i)} - 1\right) . \end{aligned}$$

For practical stability, these estimated weights $w_i$ are often clipped to a predefined range to mitigate issues arising from extremely large or small weight values, which lead to high variance in the final risk estimate.

Finally, the risk is estimated using these importance weights $w_i$ applied to the errors $e({\textbf{x}}_i)$ computed on samples ${\textbf{x}}_i$ drawn from the test part of the source distribution $g({\textbf{x}})$. The risk estimate is formulated as a standard importance-weighted average:

12 $$\begin{aligned} {\hat{R}}_{\text {Classifier}}(e, {\textbf{w}}) = \frac{1}{n_g^{\textrm{test}}} \sum _{i=1}^{n_g^{\textrm{test}}} w_i e({\textbf{x}}_i), \quad {\textbf{x}}_i \sim g({\textbf{x}}), \end{aligned}$$

This method aims to provide an unbiased estimate of the risk under the target distribution $p({\textbf{x}})$ by appropriately re-weighting observations from the source distribution $g({\textbf{x}})$.

This unbiased estimation property holds theoretically under certain conditions^3^. Notably if the support of the target distribution $p({\textbf{x}})$ is contained within the support of the source distribution $g({\textbf{x}})$. If $g({\textbf{x}})$ is zero where $p({\textbf{x}})$ is positive, the true density ratio is infinite, rendering the IW invalid for those regions.

#### Kernel mean matching (KMM)

We adopt the KMM formulation, following Huang et al.^21^, which involves solving a quadratic programming problem to estimate sample weights. We employ KMM, which is a method for bias correction that estimates the density ratio between the source distribution $g({\textbf{x}})$ and the target distribution $p({\textbf{x}})$ without directly computing the densities. It reweights the source data instances such that the weighted distribution resembles the target distribution, and, according to theory, it remains an unbiased estimate.

KMM works by finding weights $w_i$ that minimize the difference between the means of the source $g({\textbf{x}})$ and target $p({\textbf{x}})$ data distributions in the feature space defined by a kernel function. The goal is to reweight the source data so that the weighted distribution matches the target distribution.

Practical Implementation for Risk Estimation In the context of risk estimation, once the weights $w_i$ are computed using KMM, they can be incorporated into the risk estimate to adjust for the bias between the source and target distributions. The weighted risk estimate is similarly given by:

13 $$\begin{aligned} {\hat{R}}_{\text {KMM}}(e, {\textbf{w}}) = \frac{1}{n_g^{\textrm{test}}} \sum _{i=1}^{n_g^{\textrm{test}}} w_i e({\textbf{x}}_i). \end{aligned}$$

Objective Function The weights $w_i$ are computed by solving the following general optimization problem:

14 $$\begin{aligned} \min _{{\textbf{w}}} \left\| \frac{1}{n_p^{\textrm{test}}} \sum _{i=1}^{n_p^{\textrm{test}}} \phi ({\textbf{x}}_i^{\textrm{target}}) - \frac{1}{n_g^{\textrm{test}}} \sum _{j=1}^{n_g^{\textrm{test}}} w_j \phi ({\textbf{x}}_j^{\textrm{source}}) \right\| ^2 \text {s.t.}\, 0 \le w_j \le B, \quad \sum _{j=1}^{n_g^{\textrm{test}}} w_j = n_g^{\textrm{test}}, \end{aligned}$$

where $\phi ({\textbf{x}})$ represents the feature map induced by the chosen kernel (in our practical case, it will be a radial basis function), and *B* is a hyperparameter that controls the upper bound of the weights to avoid extreme values. In our study, we are not focused on clipping weights, so B is chosen to prevent this situation. To simplify the optimization, the objective function is expanded using the kernel trick. We define the necessary components using our test data partitions:

- Let $K \in {\mathbb {R}}^{n_g^{\textrm{test}} \times n_g^{\textrm{test}}}$ be the kernel matrix computed on the source test set, where each element $K_{ij} = K({\textbf{x}}_i, {\textbf{x}}_j)$ for ${\textbf{x}}_i, {\textbf{x}}_j \in D_g^{\textrm{test}}$.
- Let $\boldsymbol{\kappa } \in {\mathbb {R}}^{n_g^{\textrm{test}}}$ be a vector where each element $\kappa _i$ represents the average kernel similarity between a source test point ${\textbf{x}}_i$ and all target test points:
  $$\begin{aligned} \kappa _i = \frac{1}{n_p^{\textrm{test}}} \sum _{j=1}^{n_p^{\textrm{test}}} K({\textbf{x}}_i, {\textbf{x}}_j^{\prime }), \quad \text {where } {\textbf{x}}_i \in D_g^{\textrm{test}} \text { and } {\textbf{x}}_j^{\prime } \in D_p^{\textrm{test}}. \end{aligned}$$

With these definitions, the optimization problem to find the weights ${\textbf{w}} = (w_1, \ldots , w_{n_g^{\textrm{test}}})^\top$ can be rewritten.

15 $$\begin{aligned} \min _{\textbf{w}} \left( \frac{1}{n_{\text {target}}^2} {\textbf{w}}^\top K {\textbf{w}} - \frac{2}{n_{\text {target}}^2} \kappa ^\top {\textbf{w}} + \text {const}\right) \text {s.t.}\, 0 \le w_j \le B, \quad \sum _{j=1}^{n_g^{\textrm{test}}} w_j = n_g^{\textrm{test}} \end{aligned}$$

For a radial basis function (RBF) kernel, the kernel matrix $K$ is defined as:

16 $$\begin{aligned} K({\textbf{x}}, {\textbf{z}}) = \exp \left( -\frac{\Vert {\textbf{x}}- {\textbf{z}}\Vert ^2}{2\sigma ^2}\right) , \end{aligned}$$

where $\sigma$ is a bandwidth parameter. In practical implementations, $\sigma$ is dynamically adjusted based on the median pairwise distance between the data points:

17 $$\begin{aligned} \sigma ^2 = \frac{\text {median}\left( {\Vert {\textbf{x}}_i - {\textbf{x}}_j\Vert ^2 \mid i, j \in {1, \ldots , n_g^{\textrm{test}}}}\right) }{\log n_g^{\textrm{test}}}. \end{aligned}$$

This ensures the kernel adapts to the scale of the data, making the matching more robust across varying datasets. We will use this kernel to practically realise KMM, with the usage of the $L_2$ norm.

#### Summary of methods

We have explored several methods for estimating the target risk using samples from a source distribution, in the presence of a distribution shift $p({\textbf{x}}) \not = g({\textbf{x}})$.

- **No weighting (NW)**: This method computes the empirical average of the error directly on the source samples, yielding. It is the most straightforward approach, but it is inherently biased, as it does not account for the difference in distributions.
- **Importance weighting (IW)**: Corrects the bias by reweighting source samples using the true density ratio $p({\textbf{x}})/g({\textbf{x}})$, typically estimated via KDE. While theoretically unbiased, it suffers from high variance, especially in high dimensions where density estimation fails.
- **Classifier Mmethod**: Indirectly estimates the density ratio by training a probabilistic classifier to discriminate between source and target samples. It avoids explicit density estimation but relies on classifier calibration and can be unstable without weight clipping.
- **Kernel mean matching (KMM)**: Directly computes sample weights to minimize the discrepancy between the mean embeddings of the weighted source and target distributions in a RKHS. It bypasses explicit density or density ratio estimation, offering a more robust and stable solution, particularly in high-dimensional settings.

In essence, NW is a simple but biased baseline, IW and Classifier methods attempt bias correction via explicit or implicit density ratio estimation but face practical issues with high variance and support assumptions, while KMM tackles bias correction by matching distributions in a kernel space through weight optimization.

### Data processing

The data used in this study come from two distinct sources: artificially generated datasets and real-world observations. The artificial data allow for controlled experiments with known properties, such as specific degrees of spatial clustering, which are essential for systematically testing the limits of risk estimation methods. The real-world data, comprising ecological species occurrences and spatial layouts of immune cells from tumor microenvironments, provide critical validation in complex, practical scenarios characterized by inherent spatial biases and distribution shifts. The following subsections detail their generation and processing.

#### Artificial data

To systematically evaluate the proposed estimator, we generated synthetic datasets designed to mimic complex spatial structures and covariate shifts.

All artificial datasets were generated within a square domain ${\mathscr {X}} = [0, 100]^d$, where *d* represents the dimensionality, ranging from 2 to 4 in our experiments. For each experiment, we sampled $N=10,000$ data points for both the source $g({\textbf{x}})$ and target $p({\textbf{x}})$ distributions. This process was repeated independently 100 times for each parameter configuration to ensure statistical stability of the error metrics.

**Fig. 2:**
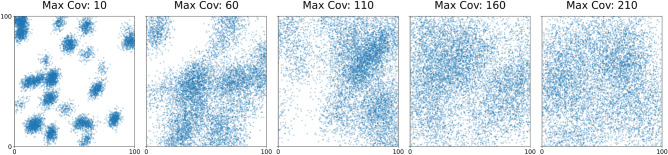
Visualizing GMM patterns with increasing maximum covariance values, transitioning from distinct clusters to a diffuse distribution.

The core of our generation process relies on Gaussian Mixture Models (GMM). To simulate complex environmental heterogeneity, we employed GMMs with 30 randomly centered components. This number was selected empirically to cover the domain space stably without creating anomalies in specific sample generations. The spread of clusters is controlled by a maximum covariance parameter ($\Sigma _{\max }$). A low $\Sigma _{\max }$ (e.g., $30-50$) results in highly clustered, distinct populations, whereas a high $\Sigma _{\max }$ (e.g., 400) or the use of a Uniform distribution results in a spread-out, diffuse structure. Figure 2 visually compares the generated GMM patterns under these different covariance constraints.

#### Robustness analysis scenarios

**Fig. 3:**
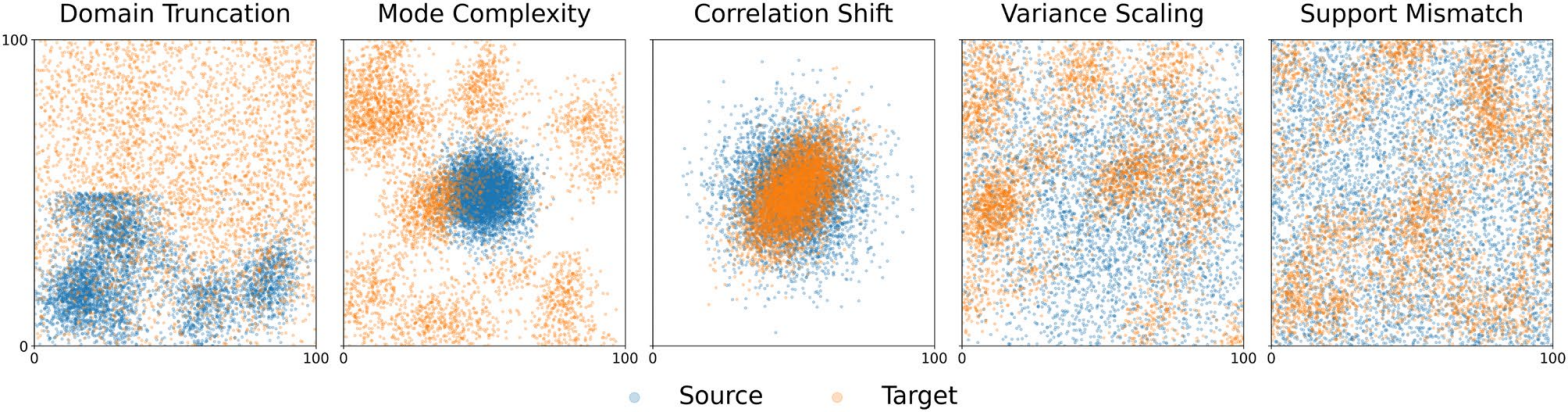
Overview of the synthetic scenarios used for robustness testing. Blue points represent the source distribution ($g({\textbf{x}})$), and orange points represent the target distribution ($p({\textbf{x}})$). These plots illustrate five representative configurations. Our experiments also include the inverse directional shifts for these categories, totaling ten scenarios.

While varying the degree of clustering provides a baseline for performance, real-world spatial data often exhibits more specific structural biases. To rigorously stress-test the KMM approach, we designed a comprehensive suite of ten synthetic scenarios, grouped into five distinct categories below. These configurations align with formal categorizations of dataset shift^22^ and are specifically tailored to reflect challenges in ecological and biological surveys, such as sampling bias, environmental dependency shifts, and scale mismatches. An overview of these patterns is visualized in Fig. 3.

The following categories summarize the key types of domain shifts modeled in our experimental framework.

- **Domain Truncation (Cropped vs. Full):**

This category examines the impact of geometric restrictions on the domain, simulating partial observability.

- The first scenario restricts the source distribution, generated as a high-clustered GMM ($\Sigma _{\max }=50$), to the lower half of the domain ($x_2 < 50$). In contrast, the target covers the full domain with lower clustering ($\Sigma _{\max }=400$). This mimics selection bias caused by accessible terrain or political borders, where the model is trained on a geographically limited subset but must generalize to the entire region.
- The second scenario reverses this configuration: the source is generated as a low-clustering GMM ($\Sigma _{\max }=400$) covering the full domain, while the target is restricted to the lower half ($x_2 < 50$) with high clustering ($\Sigma _{\max }=50$). This simulates a downscaling task where a model trained on regional data is applied to a specific local area, requiring the estimator to filter out irrelevant global information.

- **Mode Complexity Mismatch (Expansion vs. Contraction):**

This category evaluates the estimator’s behavior when the population diversity changes between domains.

- The mode expansion scenario involves a source consisting of a single centered cluster ($\mu = 50$) with a maximum covariance of 50, while the target is a multimodal GMM comprising disjoint clusters with a tighter spread ($\Sigma _{\max }=10$). This tests the model’s ability to generalize from a homogeneous training set to a diverse landscape containing multiple distinct subpopulations.
- The mode contraction scenario reverses this: the source comprises multiple scattered clusters ($\Sigma _{\max }=10$), whereas the target is a single centered mode ($\mu = 50, \Sigma _{\max }=50$). This evaluates how well a model trained on global statistics adapts to a specific local area without underperforming due to the noise present in the global dataset.

- **Correlation Structure Shift (Shift vs. Restoration):**

Here, we manipulate the covariance matrices to be either axis-aligned (diagonal) or rotated (non-diagonal) to simulate changing feature interactions.

- The correlation shift scenario starts with a source generated as a single centered component ($\mu = 50, \Sigma _{\max }=150$) constrained to a diagonal covariance matrix. The target consists of the same centered component but with a non-diagonal (rotated) covariance matrix. This tests robustness against changing environmental dependencies, such as a shift in the relationship between temperature and elevation.
- The correlation restoration scenario moves from a rotated source (non-diagonal covariance) to an axis-aligned target (diagonal covariance), with both distributions maintaining $\Sigma _{\max }=150$. This tests the method’s adaptability when complex dependencies or entangled features present in the training phase disappear or become independent in the target environment.

- **Variance Scaling (Focusing vs. Extrapolation):**

We examine shifts in the spatial spread of the data to test robustness against scale differences.

- The first case transitions from a widely dispersed source ($\Sigma _{\max }=400$) to a tightly clustered target ($\Sigma _{\max }=50$). This represents a focusing task, where a broad-scale survey is used to predict a localized phenomenon.
- The second case transitions from a tightly clustered source ($\Sigma _{\max }=50$) to a dispersed target ($\Sigma _{\max }=400$). This represents an extrapolation challenge, where the model must predict in valid regions of the domain that were sparsely sampled or entirely unseen during the training phase.

- **Support Mismatch (Structured vs. Unstructured):**

This represents an extreme covariate shift involving a Uniform distribution to test performance under severe information imbalance.

- The first scenario uses a source drawn from a uniform distribution covering the entire domain, while the target is a high-clustered GMM with $\Sigma _{\max }=50$. This requires the estimator to identify and upweight relevant signals hidden within uninformative, unstructured noise.
- The second scenario uses a high-clustered GMM source ($\Sigma _{\max }=50$) and a uniform target. This simulates the difficulty of applying a model trained on highly specific, structured data to a completely random or unexplored environment where the training structure may not apply.

These bidirectional scenarios ensure that our evaluation covers not only the magnitude of the shift but also its directionality, distinguishing between problems of interpolation, extrapolation, and structural adaptation.

#### Model Configuration

The true function $f({\textbf{x}})$ was modelled as a mixture of GMM kernels (referred to as “GMM”). For the estimation of ${\hat{f}}({\textbf{x}})$, we employed the model Gradient Boosting Regression trained on 70$\%$ of the available samples. The choice of these seemingly unconventional models was intentional, as the specific accuracy of the models is not the primary concern in this study. Instead, our focus lies on estimating the risk associated with a model error when applied to the target distribution. Squared error $e({\textbf{x}}) = \left( f({\textbf{x}}) - {\hat{f}}({\textbf{x}})\right) ^2$ served as the error metric. To evaluate the classifier-based method, we trained a Gradient Boosting Classifier using the default parameters. To ensure that the classifier was sufficiently trained, we calculated the ROC-AUC on the test set. The results are presented in Table S2 in the Supplementary Material. We prevent clipping of the weights in KMM by setting $B=1000$, which is sufficient according to the experiments shown in Fig. S5 of the Supplementary Material.

#### Real data

##### Species data Study area

The study area focuses on Finland, encompassing latitudinal and longitudinal extents that capture the primary habitats of the selected plant species. This region is characterized by boreal to subarctic climatic conditions, with a transition toward more temperate environments in southern Finland and along the coast. Prominent geographic features include numerous lakes, extensive forested zones, and coastlines along the Baltic Sea.

##### Plant occurrence

We collected occurrence data from 2000 to 2024 for several herbaceous and woody plant species native to Finland: *Tussilago farfara* L.^23^, *Anemone nemorosa L.*^24^, *Caltha palustris L.*^25^

These data were primarily obtained from the Global Biodiversity Information Facility (GBIF), which leveraged contributions from citizen science projects. We selected these species because they exhibit distinct phenological and ecological traits pertinent to boreal and subarctic ecosystems and because sufficient presence and absence records were available for the specified period (2000–2024).

##### Environmental predictors

We used 19 bioclimatic variables to model species distributions. These predictors encompass average and extreme temperature and precipitation patterns, as well as measures of climatic variability relevant to plant physiology.

To prepare the environmental data, we employed several R packages, including raster, rgdal, terra, and sf^26–29^. We standardized all spatial layers with the WGS84 coordinate reference system and then masked and cropped them to the study region, specifically Finland and relevant parts of Sweden. The final dataset was stacked into a single multi-layer raster stack for subsequent modeling.

##### Cell data

The first dataset comprises tumor biopsy images depicting various immune cell types, including conventional dendritic cells type 2 (cDC2), plasmacytoid dendritic cells (pDCs), myeloid cells, and B cells^30^. Analyzing the spatial distribution of these cells is crucial for understanding their interactions, which could lead to the identification of biomarkers for therapy response^31^. Following manual quality control, ensuring that at least half of the tissue remained intact, 78 images were selected for analysis.

Positions and types of immune cells were identified using the ImmuNet pipeline^30,32^. The tissue boundaries were detected through a segmentation algorithm implemented in the “inForm” software (v2.4.8, PerkinElmer).

Our LCF analysis, following the methodology of Martynova et al.^19^, reveals different spatial patterns among cell types: B cells exhibit high LCF values, indicating noticeable clustering, while myeloid cells show a minimal deviation from zero, reflecting a scattered distribution. A marked peak in the LCF for cDC2s suggests potential interactions at short distances.

##### Models configuration

For both datasets, we used Gradient Boosting^33^, Logistic Regression, Random Forest Classifier and MLP Classifier as our binary classification black box models. They are trained on 70$\%$ of the vailable samples. Moreover, we prevent clipping of the weights in KMM by setting $B=1000$, which is sufficient according to the experiments shown in Fig. S5 of the Supplementary Material. It is essential to perform data splitting and hyperparameter tuning to prevent overfitting on both the source and target datasets. Since our task is formulated as a black-box risk estimation problem, we are not concerned with model configuration or weight optimization, and therefore we do not perform hyperparameter tuning—we rely solely on the model’s output. It is critical to distinguish this from the random hyperparameter sampling used in our model selection experiment (Section 2.2.4); unlike tuning, which seeks an optimal model, sampling aims to generate a diverse portfolio of model behaviors to rigorously stress-test the risk estimation methods under a wide range of conditions. However, overfitted models tend to perform poorly on real-world data and introduce challenges in reliable risk estimation. We selected source-target dataset pairs to ensure meaningful transfer learning scenarios based on their inherent clustering structure. Specifically, the source dataset was chosen to exhibit a clustered structure, while the target dataset was selected to be less clustered. The degree of clustering was quantified using the area under the LCF curve. Following the recommendations of the original study, we individually selected the maximum radius values for AUC LCF for each data dimensionality and data type to obtain informative and meaningful results. Only pairs with Area under the Receiver Operating Characteristic Curve (AUC-ROC) scores exceeding 0.7 were retained, ensuring robust classification performance while avoiding overfitting.

Classification performance was evaluated using the log-loss function $e({\textbf{x}})$ for each data point ${\textbf{x}}$:

18 $$\begin{aligned} e({\textbf{x}}) = - \left( f({\textbf{x}}) \log {\hat{f}}({\textbf{x}}) + \big ( 1 - f({\textbf{x}}) \big ) \log \big ( 1 - {\hat{f}}({\textbf{x}}) \big ) \right) \end{aligned}$$

where $f({\textbf{x}}) \in \{0,1\}$ is the true label and ${\hat{f}}({\textbf{x}})$ is the predicted probability.

##### Data Preprocessing

All features were standardized to a zero mean and unit variance. We applied Principal Component Analysis (PCA) to align the feature space with our synthetic data experiments. The number of principal components was selected to preserve the intrinsic structure of the data: for clustered source datasets, components were retained to maintain separation between clusters, while for less-clustered target datasets, components were chosen to reduce redundancy without enforcing artificial structure. This approach ensured comparability with synthetic experiments while mitigating complexity. Figures S1, S5 (Supplementary material) illustrate the resulting 2D PCA projections for representative species and cell datasets, respectively, highlighting the different structures of the source (more clustered) and target (less clustered) data after dimensionality reduction.

#### Evaluation procedure

##### Comparison of methods

To evaluate the IW, we trained KDE models on train samples to approximate density ratios on test samples, ensuring theoretically grounded risk estimation. To evaluate the classifier-based method, we trained the same Gradient Boosting Classifier using the default parameters as described in Artificial data.

The performance of risk estimation was evaluated using three metrics: MAPE, Root Mean Square Error (RMSE), and Root Mean Square Percentage Error (RMSPE) across all *n* source and target pairs. These metrics allow us to assess the accuracy of risk estimation between the actual risk $R_{GT}$ and the estimated risk $R_{method}$. The subscript ‘method‘ indicates the estimation approach, which can be NW, IW, KMM, or a Classifier-based method, evaluated across various datasets and scenarios.

The three metrics are defined as follows:

19 $$\begin{aligned} \text {MAPE}&= \frac{1}{n} \sum _{i=1}^{n} \left| \frac{R_{GT}^{(i)} - R_{method}^{(i)}}{R_{GT}^{(i)}} \right| , \end{aligned}$$

20 $$\begin{aligned} \text {RMSE}&= \sqrt{\frac{1}{n} \sum _{i=1}^{n} \left( R_{GT}^{(i)} - R_{method}^{(i)} \right) ^2}, \end{aligned}$$

21 $$\begin{aligned} \text {RMSPE}&= \sqrt{\frac{1}{n} \sum _{i=1}^{n} \left( \frac{R_{GT}^{(i)} - R_{method}^{(i)}}{R_{GT}^{(i)}} \right) ^2}. \end{aligned}$$

The MAPE measure (Eq. 19) clearly explains the performance of the estimation by quantifying the average percentage deviation from a true risk. The RMSE captures the square root of the average squared differences between the estimated and true risks, focusing more on the more significant deviations. Lastly, RMSPE measures the percentage error similarly to RMSE but normalizes each difference by the true risk, allowing it to account for relative scale differences between datasets.

To better understand how the experiments were conducted across different dataset types and domain pairs, we provide a general workflow diagram. This workflow outlines the pipeline used for data generation and preprocessing, as well as the procedures for model training, validation, and risk estimation. It includes both artificial and real-world datasets (such as biological cells and species data) and demonstrates how we consistently applied the same evaluation logic across all settings.

**Fig. 4:**
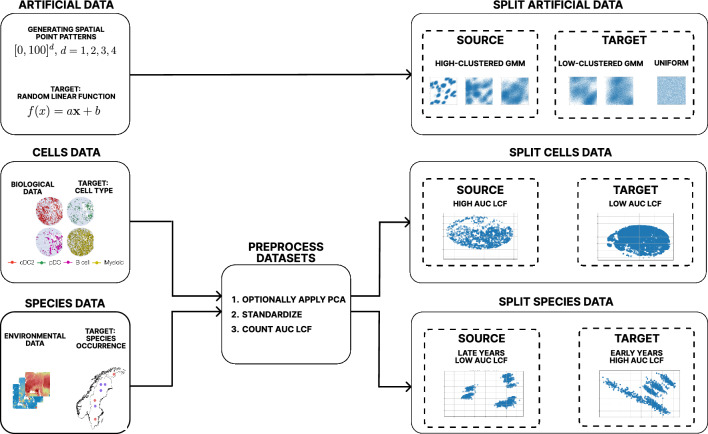
Workflow for data construction and preprocessing.

**Fig. 5:**
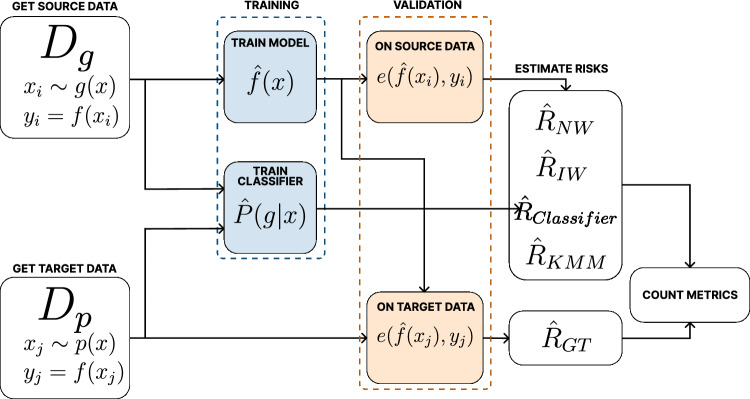
Workflow for model validation and risk estimation.

The general workflow of our experiments is divided into two parts (See Figs.  4 and  5). Figure  4 (Part A) illustrates how we construct both artificial and real datasets (cells and species data), define source and target domains, and preprocess the data including normalization, dimensionality reduction, and LCF-based splitting. Subsequently, Figure  5 (Part B) demonstrates the validation pipeline used across all dataset types: we first train a model on the source data, then a domain classifier, and finally evaluate the performance on target data using risks estimated by methods such as ${\hat{R}}_{NW}$, ${\hat{R}}_{IW}$, ${\hat{R}}_{\text {Classifier}}$, ${\hat{R}}_{\text {KMM}}$, and comparing them to $R_{GT}$.

##### Practical Demonstration of Risk Estimation for Model Selection

To demonstrate the practical significance of risk estimation, we conducted an experiment that simulates model selection under distribution shift, using previously described species and cell datasets. In real-world deployment scenarios, the true performance of a model on the target distribution is unknown. Risk estimation methods aim to provide a reliable proxy for this true risk, guiding the selection of models that are likely to perform well in the target environment. For this experiment, using species and cell datasets, we generated a pool of 200 classification models (100 Gradient Boosting, 100 Random Forest) trained on source data, with hyperparameters randomly sampled from predefined ranges (e.g. $`n\_estimators`$ [5, 20], $`max\_depth`$ [1, 4], $`learning\_rate`$ [0.01, 0.3], etc.). We filtered these models, retaining only those with a ROC AUC > 0.7 on the target test set to ensure a basic level of predictive applicability. For each retained model $m_i$, we calculated its true risk $R^{(i)}_{GT}$ and its estimated risk $R^{(i)}_{method}$ using each of the evaluated methods (NW, IW, KMM, Classifier) based on the source data. The objective was to assess how well each estimation method identifies models with low true risk using only its estimated risk $R^{(i)}_{method}$. We varied the number of selected models (*K*) to observe its impact on performance, focusing on $K=5$ for our primary analysis. Our extended results show that selecting very few models ($K=3$) introduces significant noise, reflected in high standard deviations. In contrast, selecting more models ($K=10$) leads to stabilization, with reduced variance and closer convergence between methods. The detailed results for $K=3$ and $K=10$ are provided in Supplementary Tables S3 and S4, and a comprehensive visualization of these trends is presented in Supplementary Figure S6. We applied the following model selection algorithm for each risk estimation method:

1. For each model $m_i$, determine the true risk $R^{(i)}_{GT}$ (for evaluation purposes) and the estimated risk $R^{(i)}_{method}$ using a specific risk estimation method (NW, IW, KMM, or Classifier).
2. Select the $K=5$ models with the lowest estimated risk $R^{(i)}_{method}$ according to the method. Let *A* be the set of indices of these models.
3. Compute the average true risk for the selected models and method: $R_{\text {method}} = \frac{1}{K} \sum _{i \in A} R^{(i)}_{GT}$. This value represents the actual performance of the models chosen based on the estimation method’s output.

## Results

In this section, we compare the performance of the NW, IW, and KMM methods in synthetic and real-world datasets. We take advantage of the insights and techniques derived from the analysis of synthetic data to ensure consistency and relevance in our experimental setup. A critical aspect of these experiments is the proper definition of the source and target distributions, along with a thorough clustering analysis and evaluation using appropriate metrics. Our primary focus is on scenarios involving high-clustered source data and low-clustered target data, characterized by significant distributional shifts between them.

### Artificial data

**Fig. 6:**
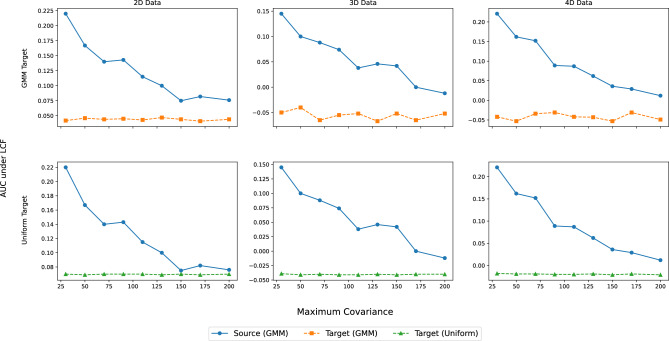
LCF analysis of clustering intensity for different covariance limits across dimensions (2D, 3D, and 4D) and target distributions (low-clustered GMM and Uniform). The single figure consolidates the six scenarios, with rows indicating the target distribution and columns representing the data dimensionality.

Initial experiments with synthetic data revealed critical insights into the limitations of IW and NW under distribution shift. We observed that lower maximum covariance values in the generation of the source GMM distribution produced stronger clustering (Fig. 2), resulting in a significant distribution shift relative to the low-clustered target GMM distribution. Quantitative analysis via LCF curves (Fig. 6) confirmed this behaviour, showing distinct clustering patterns as described in Section 2 (Methods).

A comprehensive comparison of the risk estimation methods across the proposed scenarios is presented in Table 1. The central and most consistent finding across all experimental configurations is the superior performance of the KMM method. KMM achieves the lowest error metrics in the vast majority of cases, demonstrating remarkable stability not only under varying degrees of clustering but also under geometric transformations and support mismatches.

Specifically, in scenarios involving Correlation Shift (Diagonal $\rightarrow$ Non-Diagonal) and Correlation Restoration, KMM effectively handles the rotation of the feature space, maintaining a MAPE significantly lower than NW and IW, which struggle to adapt to the changed feature dependencies. Furthermore, in the challenging Variance Scaling: Extrapolation scenario (Low Variance $\rightarrow$ High Variance), where the model must generalize to a broader domain than it was trained on, KMM outperforms density-ratio based approaches, which often exhibit unstable weights in low-density regions. Similarly, in cases of Support Mismatch (e.g., Uniform $\rightarrow$ GMM), KMM correctly identifies relevant signal within unstructured data, whereas IW frequently produces exploded error rates due to denominator instability in the density ratio estimation.

**Table 1:** Robustness evaluation of risk estimation methods under distinct structural biases and distribution shifts.

Method	2D			3D			4D		
	MAPE	RMSE	RMSPE	MAPE	RMSE	RMSPE	MAPE	RMSE	RMSPE
Domain Truncation: Cropped $\rightarrow$ Full									
NW	83.9 $\pm \!$ 1.2	1.91 $\pm \!$ 0.15	84.1 $\pm \!$ 1.3	80.6 $\pm \!$ 1.2	4.6 $\pm \!$ 0.20	80.7 $\pm \!$ 1.2	79.6 $\pm \!$ 1.1	8.9 $\pm \!$ 0.4	79.7 $\pm \!$ 1.1
IW	92.8 $\pm \!$ 1.5	2.05 $\pm \!$ 0.18	92.8 $\pm \!$ 1.4	96.2 $\pm \!$ 1.5	5.5 $\pm \!$ 0.30	96.2 $\pm \!$ 1.6	98.6 $\pm \!$ 1.6	10.9 $\pm \!$ 0.4	98.6 $\pm \!$ 1.7
KMM	**80.0** $\pm \!$ **1.0**	**1.84** $\pm \!$ **0.09**	**80.4** $\pm \!$ **1.0**	**74.2** $\pm \!$ **1.1**	**4.26** $\pm \!$ **0.15**	**74.3** $\pm \!$ **1.0**	**75.0** $\pm \!$ **0.9**	**8.4** $\pm \!$ **0.3**	**75.1** $\pm \!$ **0.9**
Classifier	89.9 $\pm \!$ 1.3	2.00 $\pm \!$ 0.16	90.0 $\pm \!$ 1.4	90.6 $\pm \!$ 1.4	5.2 $\pm \!$ 0.20	90.7 $\pm \!$ 1.4	93.9 $\pm \!$ 1.4	10.4 $\pm \!$ 0.4	93.9 $\pm \!$ 1.4
Domain Truncation: Full $\rightarrow$ Cropped									
NW	72.0 $\pm \!$ 2.0	1.07 $\pm \!$ 0.15	72.1 $\pm \!$ 2.0	92.0 $\pm \!$ 1.5	5.6 $\pm \!$ 0.30	92.0 $\pm \!$ 2.5	94.5 $\pm \!$ 1.2	10.0 $\pm \!$ 0.8	94.5 $\pm \!$ 1.5
IW	69.9 $\pm \!$ 2.2	1.04 $\pm \!$ 0.18	70.1 $\pm \!$ 2.2	75.1 $\pm \!$ 0.5	4.7 $\pm \!$ 0.15	75.1 $\pm \!$ 0.7	70.7 $\pm \!$ 3.0	7.8 $\pm \!$ 1.0	70.9 $\pm \!$ 3.0
KMM	**59.5** $\pm \!$ **3.0**	**0.92** $\pm \!$ **0.20**	**60.2** $\pm \!$ **3.0**	**72.9** $\pm \!$ **3.5**	**4.5** $\pm \!$ **0.11**	**73.2** $\pm \!$ **3.5**	**66.5** $\pm \!$ **1.5**	**7.1** $\pm \!$ **0.6**	**66.9** $\pm \!$ **1.8**
Classifier	66.4 $\pm \!$ 2.5	1.01 $\pm \!$ 0.22	66.9 $\pm \!$ 2.5	85.8 $\pm \!$ 2.0	5.2 $\pm \!$ 0.40	85.8 $\pm \!$ 2.5	82.3 $\pm \!$ 1.3	8.8 $\pm \!$ 0.7	82.6 $\pm \!$ 1.8
Mode Complexity: Expansion (Single $\rightarrow$ Clusters)									
NW	93.1 $\pm \!$ 1.3	1.41 $\pm \!$ 0.09	93.1 $\pm \!$ 1.3	90.9 $\pm \!$ 1.3	5.0 $\pm \!$ 0.20	90.9 $\pm \!$ 1.3	91.0 $\pm \!$ 1.4	12.3 $\pm \!$ 0.5	91.1 $\pm \!$ 1.4
IW	97.4 $\pm \!$ 1.4	1.47 $\pm \!$ 0.11	97.4 $\pm \!$ 1.4	99.3 $\pm \!$ 1.6	5.4 $\pm \!$ 0.30	99.3 $\pm \!$ 1.5	99.9 $\pm \!$ 1.6	13.4 $\pm \!$ 0.5	99.9 $\pm \!$ 1.6
KMM	**90.1** $\pm \!$ **0.9**	**1.37** $\pm \!$ **0.06**	**90.1** $\pm \!$ **0.9**	**85.7** $\pm \!$ **1.0**	**4.77** $\pm \!$ **0.14**	**85.8** $\pm \!$ **1.0**	**87.5** $\pm \!$ **0.8**	**11.9** $\pm \!$ **0.3**	**87.6** $\pm \!$ **0.8**
Classifier	94.6 $\pm \!$ 1.3	1.43 $\pm \!$ 0.10	94.6 $\pm \!$ 1.3	92.5 $\pm \!$ 1.4	4.9 $\pm \!$ 0.20	93.0 $\pm \!$ 1.5	98.7 $\pm \!$ 1.5	13.2 $\pm \!$ 0.5	98.7 $\pm \!$ 1.5
Mode Complexity: Contraction (Clusters $\rightarrow$ Single)									
NW	57.7 $\pm \!$ 2.0	0.58 $\pm \!$ 0.05	59.8 $\pm \!$ 2.0	59.4 $\pm \!$ 1.9	2.46 $\pm \!$ 0.12	61.5 $\pm \!$ 2.0	54.7 $\pm \!$ 1.8	4.13 $\pm \!$ 0.15	57.5 $\pm \!$ 2.0
IW	58.3 $\pm \!$ 2.0	0.60 $\pm \!$ 0.05	63.8 $\pm \!$ 2.0	92.6 $\pm \!$ 1.6	3.1 $\pm \!$ 0.20	93.1 $\pm \!$ 1.7	90.9 $\pm \!$ 1.4	5.8 $\pm \!$ 0.20	91.7 $\pm \!$ 1.5
KMM	**39.1** $\pm \!$ **1.5**	**0.43** $\pm \!$ **0.03**	**43.9** $\pm \!$ **1.7**	**43.2** $\pm \!$ **1.4**	**2.06** $\pm \!$ **0.08**	**47.8** $\pm \!$ **1.6**	**41.3** $\pm \!$ **1.2**	**3.37** $\pm \!$ **0.11**	**44.8** $\pm \!$ **1.3**
Classifier	54.2 $\pm \!$ 2.0	0.54 $\pm \!$ 0.04	58.2 $\pm \!$ 2.0	91.4 $\pm \!$ 1.6	3.13 $\pm \!$ 0.18	91.8 $\pm \!$ 1.6	91.7 $\pm \!$ 1.5	5.6 $\pm \!$ 0.20	92.2 $\pm \!$ 1.5
Correlation Shift: Diagonal $\rightarrow$ Non-Diagonal									
NW	17.5 $\pm \!$ 0.90	0.073 $\pm \!$ 0.008	19.6 $\pm \!$ 1.0	21.4 $\pm \!$ 0.9	0.22 $\pm \!$ 0.020	22.8 $\pm \!$ 0.9	27.5 $\pm \!$ 1.1	0.50 $\pm \!$ 0.030	30.2 $\pm \!$ 1.2
IW	3.2 $\pm \!$ 0.30	0.021 $\pm \!$ 0.004	3.9 $\pm \!$ 0.3	5.3 $\pm \!$ 0.4	0.069 $\pm \!$ 0.008	6.1 $\pm \!$ 0.4	7.6 $\pm \!$ 0.5	0.153 $\pm \!$ 0.012	8.9 $\pm \!$ 0.5
KMM	**2.53** $\pm \!$ **0.15**	**0.012** $\pm \!$ **0.002**	**3.2** $\pm \!$ **0.2**	**3.9** $\pm \!$ **0.2**	**0.041** $\pm \!$ **0.005**	**4.5** $\pm \!$ **0.3**	**3.4** $\pm \!$ **0.2**	**0.072** $\pm \!$ **0.006**	**4.1** $\pm \!$ **0.3**
Classifier	3.3 $\pm \!$ 0.20	0.018 $\pm \!$ 0.003	3.7 $\pm \!$ 0.3	4.1 $\pm \!$ 0.3	0.047 $\pm \!$ 0.006	4.6 $\pm \!$ 0.3	7.6 $\pm \!$ 0.4	0.219 $\pm \!$ 0.015	9.7 $\pm \!$ 0.5
Correlation Shift: Restoration (Non-Diagonal $\rightarrow$ Diagonal)									
NW	18.6 $\pm \!$ 0.9	0.123 $\pm \!$ 0.012	24.9 $\pm \!$ 1.1	46.2 $\pm \!$ 1.3	0.95 $\pm \!$ 0.05	46.2 $\pm \!$ 1.3	71.5 $\pm \!$ 1.6	3.8 $\pm \!$ 0.20	71.5 $\pm \!$ 1.6
IW	16.5 $\pm \!$ 0.9	0.115 $\pm \!$ 0.010	23.4 $\pm \!$ 1.1	46.4 $\pm \!$ 1.3	0.97 $\pm \!$ 0.05	46.5 $\pm \!$ 1.3	84.6 $\pm \!$ 1.7	4.6 $\pm \!$ 0.20	84.7 $\pm \!$ 1.7
KMM	**13.1** $\pm \!$ **0.7**	**0.081** $\pm \!$ **0.006**	**15.9** $\pm \!$ **0.7**	**28.8** $\pm \!$ **0.9**	**0.60** $\pm \!$ **0.03**	**28.8** $\pm \!$ **0.9**	**59.5** $\pm \!$ **1.1**	**3.26** $\pm \!$ **0.12**	**59.6** $\pm \!$ **1.1**
Classifier	19.9 $\pm \!$ 1.0	0.131 $\pm \!$ 0.014	20.2 $\pm \!$ 1.0	31.5 $\pm \!$ 1.0	0.68 $\pm \!$ 0.04	32.0 $\pm \!$ 1.0	67.1 $\pm \!$ 1.4	3.7 $\pm \!$ 0.20	67.2 $\pm \!$ 1.4
Support Mismatch: GMM $\rightarrow$ Uniform									
NW	23.2 $\pm \!$ 2.3	0.160 $\pm \!$ 0.018	26.1 $\pm \!$ 1.7	41.3 $\pm \!$ 1.1	0.94 $\pm \!$ 0.23	41.8 $\pm \!$ 1.0	51.0 $\pm \!$ 1.1	2.8 $\pm \!$ 0.12	39.8 $\pm \!$ 3.0
IW	21.5 $\pm \!$ 2.2	0.146 $\pm \!$ 0.021	23.9 $\pm \!$ 1.8	63.4 $\pm \!$ 0.8	1.43 $\pm \!$ 0.35	63.6 $\pm \!$ 1.1	86.6 $\pm \!$ 1.5	4.7 $\pm \!$ 0.18	86.6 $\pm \!$ 1.7
KMM	**3.5** $\pm \!$ **2.0**	**0.029** $\pm \!$ **0.014**	**4.8** $\pm \!$ **1.1**	**12.2** $\pm \!$ **2.0**	**0.35** $\pm \!$ **0.10**	**15.3** $\pm \!$ **0.9**	**30.3** $\pm \!$ **0.9**	**1.7** $\pm \!$ **0.08**	**31.1** $\pm \!$ **1.1**
Classifier	19.7 $\pm \!$ 2.2	0.147 $\pm \!$ 0.018	23.0 $\pm \!$ 1.6	33.2 $\pm \!$ 1.2	0.77 $\pm \!$ 0.28	34.0 $\pm \!$ 1.3	42.4 $\pm \!$ 1.0	2.3 $\pm \!$ 0.11	42.7 $\pm \!$ 1.2
Support Mismatch: Uniform $\rightarrow$ GMM									
NW	19.6 $\pm \!$ 0.9	0.143 $\pm \!$ 0.012	20.9 $\pm \!$ 1.0	7.6 $\pm \!$ 0.5	0.32 $\pm \!$ 0.020	10.0 $\pm \!$ 0.6	10.4 $\pm \!$ 0.5	0.88 $\pm \!$ 0.05	12.4 $\pm \!$ 0.6
IW	22.2 $\pm \!$ 1.1	0.162 $\pm \!$ 0.015	23.7 $\pm \!$ 1.1	13.7 $\pm \!$ 0.7	0.50 $\pm \!$ 0.040	16.5 $\pm \!$ 0.8	12.4 $\pm \!$ 0.7	1.09 $\pm \!$ 0.06	16.6 $\pm \!$ 0.8
KMM	**16.9** $\pm \!$ **0.8**	**0.128** $\pm \!$ **0.010**	**18.8** $\pm \!$ **0.8**	**7.2** $\pm \!$ **0.3**	**0.262** $\pm \!$ **0.015**	**9.0** $\pm \!$ **0.5**	**4.2** $\pm \!$ **0.3**	**0.40** $\pm \!$ **0.03**	**6.2** $\pm \!$ **0.4**
Classifier	26.0 $\pm \!$ 1.2	0.180 $\pm \!$ 0.016	27.0 $\pm \!$ 1.3	31.1 $\pm \!$ 1.2	0.97 $\pm \!$ 0.050	32.1 $\pm \!$ 1.2	35.1 $\pm \!$ 1.3	2.50 $\pm \!$ 0.11	37.5 $\pm \!$ 1.3

The table compares performance metrics (MAPE, RMSE, RMSPE) across 2D, 3D, and 4D dimensions for different synthetic scenarios designed to stress-test estimator stability (e.g., domain truncation, correlation shifts, and mode mismatches). The best-performing method per block (lowest MAPE) is in bold.

To provide a more granular analysis of this behavior, we systematically evaluated risk estimation performance across a range of maximum covariance limits for GMM source distribution, which directly control the intensity of the covariate shift. The results of these experiments are presented for dimensions 2D, 3D, and 4D. Figure S3 details the performance metrics for the uniform target distribution, while Figure S4 shows the corresponding results for the GMM target distribution.

### Real data

We present experiments conducted on real-world datasets, which are critical for understanding the challenges and effectiveness of risk estimation in spatial modeling. Real data often exhibit greater complexity and variability than synthetic datasets, enabling us to evaluate our models in realistic scenarios.

A comprehensive comparison of the risk estimation methods on these real datasets (reduced to 2D, 3D, and 4D via PCA) is presented in Table 2. Consistent with our findings from the artificial scenarios, the KMM method achieves the lowest error metrics in the vast majority of cases. For instance, in the 4D Immune cells data, KMM maintains a manageable MAPE ranging from 54.3 to 93.4% depending on the predictive model. In contrast, IW fails to produce stable estimates, yielding significantly higher MAPE values ranging from 101.9 to 544.6%. This demonstrates that KMM is the most robust and reliable method for risk estimation under spatial distribution shift, effectively overcoming the limitations of density-ratio-based approaches.

**Table 2:** Performance comparison of NW, IW, KMM, and Classifier-based risk estimation methods across PCA dimensions (2D, 3D, 4D) for real data.

Method	2D			3D			4D		
	MAPE	RMSE	RMSPE	MAPE	RMSE	RMSPE	MAPE	RMSE	RMSPE
Species Gradient Boosting Model									
NW	67.5 $\pm \!$ 3.0	0.98 $\pm \!$ 0.07	70.0 $\pm \!$ 3.0	53.1 $\pm \!$ 2.0	0.91 $\pm \!$ 0.05	60.2 $\pm \!$ 2.5	47.5 $\pm \!$ 2.0	0.72 $\pm \!$ 0.05	54.2 $\pm \!$ 2.5
IW	88.6 $\pm \!$ 4.0	1.16 $\pm \!$ 0.08	89.0 $\pm \!$ 4.0	91.3 $\pm \!$ 4.0	1.13 $\pm \!$ 0.08	91.5 $\pm \!$ 4.0	95.1 $\pm \!$ 4.0	1.00 $\pm \!$ 0.08	95.3 $\pm \!$ 4.0
KMM	**44.6** $\pm \!$ **2.0**	**0.78** $\pm \!$ **0.02**	**50.9** $\pm \!$ **2.0**	**39.8** $\pm \!$ **1.0**	**0.801** $\pm \!$ **0.003**	**49.7** $\pm \!$ **2.0**	**33.5** $\pm \!$ **1.5**	**0.61** $\pm \!$ **0.01**	**43.5** $\pm \!$ **2.0**
Classifier	52.6 $\pm \!$ 2.5	0.83 $\pm \!$ 0.02	57.5 $\pm \!$ 2.5	51.1 $\pm \!$ 1.3	0.811 $\pm \!$ 0.005	56.9 $\pm \!$ 1.5	44.4 $\pm \!$ 1.8	0.63 $\pm \!$ 0.01	52.2 $\pm \!$ 2.5
Species Logistic Regression Model									
NW	60.1 $\pm \!$ 3.0	1.03 $\pm \!$ 0.08	63.4 $\pm \!$ 3.0	62.1 $\pm \!$ 3.0	1.01 $\pm \!$ 0.08	65.0 $\pm \!$ 3.0	44.8 $\pm \!$ 2.0	0.41 $\pm \!$ 0.03	42.9 $\pm \!$ 2.0
IW	88.5 $\pm \!$ 4.0	1.28 $\pm \!$ 0.10	89.1 $\pm \!$ 4.0	92.2 $\pm \!$ 4.0	1.30 $\pm \!$ 0.10	92.5 $\pm \!$ 4.0	89.9 $\pm \!$ 4.0	0.79 $\pm \!$ 0.06	90.0 $\pm \!$ 4.0
KMM	**36.9** $\pm \!$ **1.8**	**0.78** $\pm \!$ **0.01**	**45.2** $\pm \!$ **1.0**	**37.8** $\pm \!$ **2.0**	**0.75** $\pm \!$ **0.02**	**45.2** $\pm \!$ **2.0**	**34.3** $\pm \!$ **1.5**	**0.33** $\pm \!$ **0.01**	**41.2** $\pm \!$ **0.3**
Classifier	41.3 $\pm \!$ 2.0	0.82 $\pm \!$ 0.02	48.0 $\pm \!$ 1.2	46.9 $\pm \!$ 2.5	0.81 $\pm \!$ 0.03	52.8 $\pm \!$ 2.5	39.2 $\pm \!$ 1.8	0.41 $\pm \!$ 0.03	41.8 $\pm \!$ 0.3
Species Random Forest Model									
NW	83.8 $\pm \!$ 4.0	3.85 $\pm \!$ 0.03	86.4 $\pm \!$ 4.0	67.6 $\pm \!$ 3.0	1.63 $\pm \!$ 0.02	74.2 $\pm \!$ 3.5	63.1 $\pm \!$ 3.0	1.99 $\pm \!$ 0.02	69.8 $\pm \!$ 3.5
IW	92.6 $\pm \!$ 4.5	3.90 $\pm \!$ 0.05	93.1 $\pm \!$ 4.5	92.1 $\pm \!$ 4.5	1.72 $\pm \!$ 0.06	92.5 $\pm \!$ 4.5	96.4 $\pm \!$ 4.5	2.07 $\pm \!$ 0.06	96.5 $\pm \!$ 4.5
KMM	**70.7** $\pm \!$ **1.5**	**3.775** $\pm \!$ **0.004**	**79.6** $\pm \!$ **0.5**	**52.1** $\pm \!$ **2.5**	**1.59** $\pm \!$ **0.01**	**66.3** $\pm \!$ **2.0**	**49.5** $\pm \!$ **2.0**	**1.944** $\pm \!$ **0.003**	**61.6** $\pm \!$ **1.5**
Classifier	74.7 $\pm \!$ 1.8	3.782 $\pm \!$ 0.004	80.8 $\pm \!$ 0.5	61.5 $\pm \!$ 3.0	1.61 $\pm \!$ 0.01	70.9 $\pm \!$ 2.5	56.8 $\pm \!$ 2.5	1.949 $\pm \!$ 0.003	65.3 $\pm \!$ 2.0
Species Neural Network Model									
NW	40.1 $\pm \!$ 2.0	0.60 $\pm \!$ 0.04	48.5 $\pm \!$ 2.5	45.5 $\pm \!$ 2.0	0.81 $\pm \!$ 0.06	52.3 $\pm \!$ 2.5	42.3 $\pm \!$ 2.0	0.50 $\pm \!$ 0.03	46.8 $\pm \!$ 2.0
IW	85.2 $\pm \!$ 4.0	1.01 $\pm \!$ 0.08	88.2 $\pm \!$ 4.0	90.3 $\pm \!$ 4.5	1.21 $\pm \!$ 0.09	92.6 $\pm \!$ 4.5	95.2 $\pm \!$ 4.5	0.91 $\pm \!$ 0.07	95.8 $\pm \!$ 4.5
KMM	**30.6** $\pm \!$ **1.5**	**0.45** $\pm \!$ **0.03**	**38.3** $\pm \!$ **2.0**	**33.1** $\pm \!$ **1.5**	**0.66** $\pm \!$ **0.05**	**41.5** $\pm \!$ **2.0**	**32.9** $\pm \!$ **1.5**	**0.35** $\pm \!$ **0.04**	**36.9** $\pm \!$ **1.8**
Classifier	35.3 $\pm \!$ 1.8	0.51 $\pm \!$ 0.03	42.5 $\pm \!$ 2.0	38.4 $\pm \!$ 1.8	0.76 $\pm \!$ 0.05	45.9 $\pm \!$ 2.2	37.5 $\pm \!$ 1.8	0.43 $\pm \!$ 0.04	40.8 $\pm \!$ 2.0
Immune cells Gradient Boosting Model									
NW	47.2 $\pm \!$ 2.5	0.89 $\pm \!$ 0.03	54.5 $\pm \!$ 2.0	91.2 $\pm \!$ 4.0	16.1 $\pm \!$ 0.8	97.5 $\pm \!$ 4.5	84.5 $\pm \!$ 2.0	16.2 $\pm \!$ 0.3	88.9 $\pm \!$ 1.5
IW	55.2 $\pm \!$ 3.0	0.93 $\pm \!$ 0.05	60.8 $\pm \!$ 2.5	242.0 $\pm \!$ 10.0	151.0 $\pm \!$ 10.0	1229.0 $\pm \!$ 50.0	101.9 $\pm \!$ 5.0	36.5 $\pm \!$ 1.5	257.8 $\pm \!$ 10.0
KMM	**40.8** $\pm \!$ **2.0**	**0.83** $\pm \!$ **0.01**	**50.3** $\pm \!$ **1.5**	**81.3** $\pm \!$ **3.5**	**15.2** $\pm \!$ **0.5**	**88.6** $\pm \!$ **4.0**	**79.9** $\pm \!$ **1.5**	**15.5** $\pm \!$ **0.19**	**85.6** $\pm \!$ **1.0**
Classifier	46.9 $\pm \!$ 2.5	0.85 $\pm \!$ 0.01	53.6 $\pm \!$ 1.8	91.1 $\pm \!$ 4.0	15.9 $\pm \!$ 0.2	95.7 $\pm \!$ 4.3	84.4 $\pm \!$ 2.0	16.1 $\pm \!$ 0.3	88.3 $\pm \!$ 1.2
Immune cells Logistic Regression Model									
NW	53.0 $\pm \!$ 2.5	2.27 $\pm \!$ 0.04	64.7 $\pm \!$ 3.0	88.4 $\pm \!$ 2.5	1.01 $\pm \!$ 0.02	88.6 $\pm \!$ 2.5	90.9 $\pm \!$ 3.0	1.13 $\pm \!$ 0.02	91.0 $\pm \!$ 3.0
IW	57.2 $\pm \!$ 3.0	2.27 $\pm \!$ 0.04	65.2 $\pm \!$ 3.0	653.2 $\pm \!$ 25.0	1.61 $\pm \!$ 0.30	203.4 $\pm \!$ 10.0	135.0 $\pm \!$ 5.0	2.11 $\pm \!$ 0.50	364.0 $\pm \!$ 15.0
KMM	**42.9** $\pm \!$ **2.0**	**2.18** $\pm \!$ **0.03**	**58.0** $\pm \!$ **2.5**	**83.2** $\pm \!$ **0.3**	**0.963** $\pm \!$ **0.005**	**83.5** $\pm \!$ **0.5**	**84.6** $\pm \!$ **0.3**	**1.075** $\pm \!$ **0.004**	**84.9** $\pm \!$ **0.3**
Classifier	52.7 $\pm \!$ 2.5	2.29 $\pm \!$ 0.05	63.9 $\pm \!$ 3.0	84.6 $\pm \!$ 0.6	0.977 $\pm \!$ 0.006	84.8 $\pm \!$ 0.6	85.3 $\pm \!$ 0.3	1.087 $\pm \!$ 0.006	85.5 $\pm \!$ 0.3
Immune cells Random Forest Model									
NW	46.2 $\pm \!$ 2.0	1.11 $\pm \!$ 0.03	53.8 $\pm \!$ 2.5	52.3 $\pm \!$ 2.5	2.45 $\pm \!$ 0.05	57.3 $\pm \!$ 3.0	70.4 $\pm \!$ 3.0	4.76 $\pm \!$ 0.06	72.6 $\pm \!$ 3.5
IW	52.3 $\pm \!$ 2.5	1.13 $\pm \!$ 0.03	57.4 $\pm \!$ 3.0	64.3 $\pm \!$ 3.0	3.41 $\pm \!$ 0.50	197.1 $\pm \!$ 10.0	149.3 $\pm \!$ 8.0	14.9 $\pm \!$ 1.0	375.6 $\pm \!$ 15.0
KMM	**35.5** $\pm \!$ **1.5**	**1.02** $\pm \!$ **0.03**	**46.7** $\pm \!$ **2.0**	**28.3** $\pm \!$ **1.0**	**2.34** $\pm \!$ **0.01**	**38.9** $\pm \!$ **1.5**	**54.3** $\pm \!$ **2.5**	**4.63** $\pm \!$ **0.02**	**59.5** $\pm \!$ **3.0**
Classifier	44.4 $\pm \!$ 2.0	1.08 $\pm \!$ 0.03	52.1 $\pm \!$ 2.5	34.1 $\pm \!$ 1.5	2.37 $\pm \!$ 0.01	42.4 $\pm \!$ 2.0	59.6 $\pm \!$ 2.5	4.66 $\pm \!$ 0.02	63.0 $\pm \!$ 3.0
Immune cells Neural Network Model									
NW	35.3 $\pm \!$ 1.8	0.50 $\pm \!$ 0.01	43.2 $\pm \!$ 2.0	98.9 $\pm \!$ 0.2	12.55 $\pm \!$ 0.05	99.0 $\pm \!$ 0.1	97.6 $\pm \!$ 1.0	17.97 $\pm \!$ 0.35	97.7 $\pm \!$ 1.0
IW	41.5 $\pm \!$ 2.0	0.52 $\pm \!$ 0.01	48.0 $\pm \!$ 2.5	327.1 $\pm \!$ 15.0	43.2 $\pm \!$ 2.0	926.1 $\pm \!$ 40.0	544.6 $\pm \!$ 25.0	167.3 $\pm \!$ 10.0	202.5 $\pm \!$ 10.0
KMM	**23.6** $\pm \!$ **1.2**	**0.39** $\pm \!$ **0.05**	**32.1** $\pm \!$ **1.5**	**97.1** $\pm \!$ **0.5**	**12.488** $\pm \!$ **0.005**	**97.3** $\pm \!$ **0.2**	**93.4** $\pm \!$ **0.8**	**17.23** $\pm \!$ **0.15**	**93.5** $\pm \!$ **0.8**
Classifier	31.4 $\pm \!$ 1.5	0.49 $\pm \!$ 0.01	41.5 $\pm \!$ 2.0	98.5 $\pm \!$ 0.1	12.504 $\pm \!$ 0.005	98.7 $\pm \!$ 0.1	95.1 $\pm \!$ 0.8	17.59 $\pm \!$ 0.16	95.43 $\pm \!$ 0.8

KMM method consistently outperforms all other approaches across the majority of datasets and dimensionalities. The best-performing method per block (lowest MAPE) is in bold.

#### Species data

The first dataset contained information on various plant species, with features including longitude, latitude, and climate factors. The target variable for our prediction task was the presence or absence of a given species.

For the source-target separation, we focused on temporal modeling. Specifically, our goal was to assess the risk in a less clustered distribution of data while anticipating that future data will be more spatially dispersed. To achieve this, we divide the data based on early and late years, enabling us to estimate the risk in the target distribution for the binary classification task.

We validated our framework on the clustered source and less-clustered target datasets, preprocessed and dimensionally reduced as described in the Section 2. The degree of clustering, measured through the area of the LCF curve, confirmed the structural distinction between the source and target datasets. For this classification, we have chosen *Tussilago farfara L.*, *Caltha palustris L.*, and *Anemone nemorosa L.* due to the appropriate LCF for source and target splitting and the presence of both classes. Critically, when IW performed poorly relative to NW, we observed a replication of the synthetic data problem - highlighting sensitivity to distributional mismatch. Quantitative results (Table 2) demonstrate the consistency of our risk estimation metrics (MAPE, RMSE, RMSPE) across domain shifts, reinforcing the robustness of the KMM approach for exact dimensions as in artificial data.

In addition to the dimension reduction via PCA, we applied the proposed risk estimators (NW, IW, KMM, Classifier) directly to the original high-dimensional feature space. These results are presented in Table 3.

#### Immune cell data

The second dataset originates from a study on immune cells. This dataset comprises the positional and biological features of four distinct types of immune cells. For our task, we split the dataset to conduct a binary classification of the cell types. We have chosen B-cells and myeoild cells for this classification due to an appropriate LCF for source and target splitting. Similarly, after using the same preprocessing, we selected less clustered data for the target distribution and more clustered data for the source distribution. The example was demonstrated in Fig. S2.

We evaluated the performance of the binary classification task for different dimensions, analogous to our experiments on plant species data. The results, provided in Table 2, generally mirror the species data findings. Extreme mismatches in higher dimensions (4D) caused significant instability in IW-based estimation, further validating KMM’s superior constraint handling. As with the species data, we also evaluated performance on the full, non-reduced feature set; these results are detailed in Table 3, showing even more pronounced differences between KMM and density-ratio methods in the original high-dimensional space.

**Table 3:** Performance comparison of NW, IW, KMM, and Classifier-based risk estimation methods across original datasets (all features) for different data types.

Model type	Species dataset				Immune cells dataset			
	NW	IW	KMM	Classifier	NW	IW	KMM	Classifier
Gradient Boosting								
MAPE	71.9 $\pm \!$ 3.5	174.6 $\pm \!$ 8.0	**39.5** $\pm \!$ **2.0**	49.2 $\pm \!$ 2.5	102.5 $\pm \!$ 5.0	6686 $\pm \!$ 300	**66.4** $\pm \!$ **3.0**	79.4 $\pm \!$ 3.5
RMSE	0.90 $\pm \!$ 0.04	3.00 $\pm \!$ 0.15	**0.53** $\pm \!$ **0.01**	0.76 $\pm \!$ 0.02	18.24 $\pm \!$ 0.90	716 $\pm \!$ 35	**12.27** $\pm \!$ **0.15**	12.65 $\pm \!$ 0.18
RMSPE	74.1 $\pm \!$ 3.5	282.1 $\pm \!$ 12.0	**46.9** $\pm \!$ **2.2**	57.0 $\pm \!$ 2.8	116.0 $\pm \!$ 5.5	4912 $\pm \!$ 250	**76.7** $\pm \!$ **3.5**	87.5 $\pm \!$ 4.0
Logistic Regression								
MAPE	192.0 $\pm \!$ 9.0	543.4 $\pm \!$ 25.0	**38.41** $\pm \!$ **0.15**	38.71 $\pm \!$ 0.18	91.8 $\pm \!$ 4.5	1611 $\pm \!$ 80	**60.3 ** $\pm \!$ **1.0**	63.0 $\pm \!$ 1.2
RMSE	2.96 $\pm \!$ 0.15	10.00 $\pm \!$ 0.50	**0.31** $\pm \!$ **0.02**	0.36 $\pm \!$ 0.02	0.64 $\pm \!$ 0.03	70.6 $\pm \!$ 3.5	**0.527 ** $\pm \!$ **0.006**	0.543 $\pm \!$ 0.007
RMSPE	355.0 $\pm \!$ 18.0	1372.6 $\pm \!$ 70.0	**42.20** $\pm \!$ **0.03**	42.28 $\pm \!$ 0.04	131.9 $\pm \!$ 6.0	1923 $\pm \!$ 95	**65.** $\pm \!$ **0.6**	66.8 $\pm \!$ 0.7
Random Forest								
MAPE	82.0 $\pm \!$ 4.0	261.5 $\pm \!$ 13.0	**62.3** $\pm \!$ **0.5**	64.1 $\pm \!$ 0.6	129.8 $\pm \!$ 6.0	661.8 $\pm \!$ 30.0	**85.** $\pm \!$ **0.2**	86.5 $\pm \!$ 0.3
RMSE	1.08 $\pm \!$ 0.05	5.69 $\pm \!$ 0.25	**0.87** $\pm \!$ **0.03**	1.02 $\pm \!$ 0.04	14.32 $\pm \!$ 0.70	412.2 $\pm \!$ 20.0	**14.284** $\pm \!$ **0.007**	14.301 $\pm \!$ 0.008
RMSPE	83.4 $\pm \!$ 4.0	547.0 $\pm \!$ 25.0	**67.8** $\pm \!$ **1.0**	70.8 $\pm \!$ 1.2	241.3 $\pm \!$ 12.0	1412 $\pm \!$ 70	**91.5** $\pm \!$ **1.2**	94.8 $\pm \!$ 1.5
Neural Network								
MAPE	80.1 $\pm \!$ 4.0	251.8 $\pm \!$ 12.0	**34.6** $\pm \!$ **0.8**	36.7 $\pm \!$ 0.9	122.7 $\pm \!$ 6.0	6618 $\pm \!$ 300	**71.3** $\pm \!$ **1.5**	75.3 $\pm \!$ 1.8
RMSE	1.03 $\pm \!$ 0.05	5.12 $\pm \!$ 0.25	**0.281** $\pm \!$ **0.015**	0.321 $\pm \!$ 0.015	14.53 $\pm \!$ 0.70	540.8 $\pm \!$ 25.0	**13.00** $\pm \!$ **0.07**	13.17 $\pm \!$ 0.08
RMSPE	85.5 $\pm \!$ 4.0	504.0 $\pm \!$ 25.0	**37.9** $\pm \!$ **0.5**	39.3 $\pm \!$ 0.6	186.3 $\pm \!$ 9.0	8321 $\pm \!$ 400	**79.38** $\pm \!$ **0.15**	79.76 $\pm \!$ 0.18

### Practical demonstration of risk estimation for model selection

The values of $R_{\text {selected}}$ for each method, dataset, and dimensionality are presented in Table 4. As shown in the table, the KMM method consistently selected model sets with the lowest average true risk across various data configurations and tasks (species and cells). This demonstrates its effectiveness in the practical task of identifying models likely to perform best when deployed in a target domain with distribution shift, highlighting the value of accurate risk estimation.

**Table 4:** Average true risk ($R_{\text {selected}}$) for sets of $K=5$ models (values multiplied by 1000).

Data type	Dimensionality	NW	IW	KMM	Classifier
Species	2D	2.335 $\pm \!$ 0.18	2.663 $\pm \!$ 0.20	**1.549** $\pm \!$ **0.12**	2.232 $\pm \!$ 0.17
	3D	4.539 $\pm \!$ 0.29	6.023 $\pm \!$ 0.35	**1.848** $\pm \!$ **0.14**	4.204 $\pm \!$ 0.26
	4D	5.896 $\pm \!$ 0.34	7.499 $\pm \!$ 0.41	**2.892** $\pm \!$ **0.21**	4.806 $\pm \!$ 0.28
	ALL	11.594 $\pm \!$ 0.52	12.381 $\pm \!$ 0.58	**8.220** $\pm \!$ **0.36**	10.044 $\pm \!$ 0.44
Immune cells	2D	2.478 $\pm \!$ 0.16	4.182 $\pm \!$ 0.27	**2.233** $\pm \!$ **0.14**	2.376 $\pm \!$ 0.15
	3D	4.346 $\pm \!$ 0.24	5.011 $\pm \!$ 0.29	**3.591** $\pm \!$ **0.19**	4.190 $\pm \!$ 0.23
	4D	4.952 $\pm \!$ 0.28	5.406 $\pm \!$ 0.30	**3.980** $\pm \!$ **0.22**	5.160 $\pm \!$ 0.25
	ALL	6.848 $\pm \!$ 0.37	9.418 $\pm \!$ 0.49	**5.258** $\pm \!$ **0.28**	6.189 $\pm \!$ 0.33

### Comparison and analysis

We show that KMM outperforms traditional methods and effectively addresses the issues caused by poor IW with KDE. This improvement is particularly evident in the analysis of both artificial and real datasets. KMM’s superior performance is most noticeable in situations where KDE struggles to estimate weights accurately, leading to biased results. KMM successfully mitigates these problems by adjusting sample weights more effectively, leading to better risk estimation and overall model performance. To ensure that our comparison of risk estimators is meaningful, we first validated the performance of these models. Table S1 summarizes the ROC AUC scores for the four different predictive models used in our experiments. We exclusively utilized models achieving an ROC AUC score greater than 0.7.

Tables 1 and 2 present a comprehensive comparison of four risk estimation methods: NW, IW, KMM, and Classifier. Performance is evaluated using three metrics: MAPE, RMSE, and RMSPE. The results cover distinct experimental scenarios: nine synthetic scenarios designed to stress-test estimator stability (Table 1) and real-world datasets (Species and Immune cells) with varying PCA dimensionality (Table 2).

In the artificial data experiments, particularly in scenarios involving Support Mismatch (e.g., Uniform vs. GMM) and Variance Scaling, KMM consistently demonstrates superior performance across all dimensions and metrics. For instance, in the Extrapolation scenario (Low Variance $\rightarrow$ High Variance), KMM achieves an MAPE substantially lower than both NW and IW. This pattern persists in geometric shifts such as Domain Truncation, although the absolute error values naturally vary with the complexity of the shift. Notably, while NW and IW show substantial degradation in high-variance or unstructured settings, KMM maintains relatively better performance, indicating its greater robustness to structural distribution mismatches.

Similar patterns emerge in the Mode Complexity and Correlation Shift scenarios, where KMM consistently outperforms alternative methods. This demonstrates KMM’s effectiveness in handling more complex, multi-modal, and rotated target distributions where density estimation becomes unstable.

As illustrated in Figs. S3 and S4, KMM consistently outperforms NW, IW, and the classifier-based approach across all metrics, regardless of the severity of the shift controlled by the maximum covariance parameter. These figures also highlight a critical weakness in the IW method. As the source data become more strongly clustered (lower covariance), the magnitude of the covariate shift increases. Consequently, the performance of IW deteriorates sharply, whereas KMM maintains robust and superior performance, demonstrating its effectiveness in scenarios where traditional methods fail.

To further illustrate this phenomenon, Figure S5 in the Supplementary Material shows the distribution of IW weights for both real and artificial data compared to KMM. This figure confirms that IW weights are frequently much larger than those of KMM, as described in the Methods section, providing direct evidence of the “exploding” weights effect.

The experiments with real-world datasets (Table 2) further validate KMM’s superiority. In the species data, KMM reduces the MAPE significantly compared to NW and by an even larger margin compared to IW across all dimensionalities. The Immune cells data presents the most challenging scenario, with high baseline errors for all methods. Here, KMM’s advantage is particularly striking, dramatically reducing MAPE compared to both NW and IW, especially in the 4D case, where IW fails with an extremely high error rate.

Table 3 extends our analysis to the full-dimensional feature space without dimensionality reduction through PCA. This table compares the same methods on the two real-world datasets using the same three performance metrics. The results further emphasize KMM’s robustness and effectiveness in high-dimensional spaces.

In the Species dataset with all features, KMM achieves a MAPE significantly lower than both NW and IW. The improvement is even more dramatic for RMSE, where KMM shows a substantial reduction compared to NW and an even larger reduction compared to IW. This pattern is consistent across all metrics. The Immune Cells dataset presents an even more challenging scenario across the entire feature space, with NW and IW methods exhibiting extremely high error rates. In stark contrast, KMM maintains remarkable stability.

These results collectively demonstrate that KMM consistently provides more accurate risk estimation across various datasets, dimensionalities, and structural shifts. Its superior performance is particularly evident in challenging scenarios involving high dimensionality or complex geometric mismatches, where traditional methods, such as IW with KDE, frequently fail catastrophically. The robustness of KMM to the curse of dimensionality and its ability to handle complex, real-world data distributions make it a preferable choice for covariate shift adaptation in practical applications.

## Discussion

Although sample reweighting is asymptotically unbiased, it often proves inaccurate for finite sample datasets, particularly when sample selection bias is substantial, as demonstrated in the classifier analysis by Liu et al.^34^. For example, as shown in Table 1 under the Support Mismatch scenario (GMM $\rightarrow$ Uniform) in 4D, IW yields an MAPE of 86.6% compared to KMM’s 30.3%. This performance gap stems from IW’s reliance on KDE. As dimensionality increases, KDE requires exponentially more samples to maintain accuracy, which directly impacts IW’s weight estimates. KMM avoids this issue by reweighting samples to minimize the MMD between distributions in an RKHS. Critically, MMD can be estimated with $O(1/\sqrt{n})$ error without explicit density estimation. This explains KMM’s robustness in high dimensions, while IW fails catastrophically (for instance, Immune Cells 4D MAPE > 500% in Table 2).

The practical implication is clear: for spatial modeling tasks where environmental covariates naturally create high-dimensional feature spaces, KMM provides the only reliable risk estimates among the methods tested. This explains its strong performance on the Species dataset (Table 3), where incorporating multiple climate variables would typically exacerbate IW’s instability.

Both IW and classifier-based methods share a critical vulnerability: they depend on estimating the density ratio $p({\textbf{x}})/g({\textbf{x}})$, albeit through different approaches. IW fails when the source distribution $g({\textbf{x}})$ is underestimated in sparse regions, for example, as it was shown in our case (Fig. S1). Although classifier-based methods outperform IW in our experiments (for example, reducing Species Logistic Regression MAPE from 543.37 to 38.71%), they suffer from inherent limitations. As Bickel et al. (2009)^3^ demonstrate, these methods prioritize discriminative accuracy over density ratio estimation, often producing miscalibrated probabilities when classifiers overfit to dataset-specific artifacts. In the 4D immune cells setting in Table 2), classifier-based risk estimates consistently trail KMM, with gaps ranging from 0.7 to 5.3 MAPE across all models, averaging about 3.1 MAPE. This gap occurs because classifiers optimize for discriminative accuracy^3^ rather than density ratio calibration. When classifiers overfit to biased or non-generalizable patterns in the training data (for instance, spurious spatial correlations caused by sampling imbalances), their probability estimates become poorly calibrated—a flaw KMM circumvents by directly matching distributions in kernel space.

This suggests that while classifier-based approaches are a useful heuristic, they cannot match KMM’s theoretical guarantees. The latter’s direct minimization of MMD provides a principled alternative that aligns with recent work on robust predictive inference^35^, although we focus on risk estimation rather than their conformal prediction framework.

While KMM demonstrated superior performance in our study, several limitations should be acknowledged. An important implication of our decision not to perform hyperparameter tuning on predictive models is that it strengthens the robustness of our comparative findings. Since all risk estimation methods (NW, IW, KMM, Classifier) were evaluated on the same set of untuned models, the consistent outperformance of KMM across this diverse range of model behaviors underscores its superiority as a risk estimation technique, independent of model optimization. Although tuning would likely improve the absolute predictive performance of the models, we expect the relative ranking of the risk estimation methods to remain unchanged. This concern is mitigated by our model selection experiment (Section “Evaluation procedure”), which demonstrated that KMM’s superiority was consistent across a wide pool of 200 models with varying performance, confirming the robustness of our comparative findings.

The effectiveness of KMM, like all kernel-based methods, is highly dependent on the choice of the kernel function and its hyperparameters. An inadequately chosen kernel may fail to capture the complex relationships within the data, leading to suboptimal weight estimation and less accurate risk assessments. Our analysis relied on a standard kernel with a common heuristic for parameter selection, but a more exhaustive search or adaptive selection process could potentially yield further improvements, representing an avenue for future work.

Moreover, KMM faces computational challenges with very large datasets. The core of the method involves the computation of a Gram matrix, which scales quadratically with the number of samples. While techniques like random Fourier features or divide-and-conquer approaches can improve scalability, these were not employed in our study. As a result, applying KMM to massive spatial datasets may require these more advanced computational strategies. We have shown these tendencies in Supplementary Figure S7, which illustrates the computational cost of this method as a function of sample size. The plot clearly shows the super-linear, polynomial growth in KMM’s evaluation time on Species data.

Finally, our study focuses on methods that correct for distribution shift primarily through sample reweighting. However, a separate class of “doubly robust” estimators exists that combines IW with a regression-based component (a control functional) to simultaneously correct for sampling bias and reduce variance. As demonstrated by^36^, such doubly robust estimators can achieve superior performance, especially in complex settings where samples are biased and affected by noise, conditions often encountered in real-world spatial analysis. By focusing only on reweighting techniques, our study may overlook these potentially more powerful and stable estimators, marking a clear direction for future comparative studies.

Spatial data amplifies conventional distribution shift problems through two mechanisms: (1) inherent clustering due to environmental gradients (evidenced by LCF curves in Fig. 6), and (2) sampling biases where certain regions are overrepresented in source data. Traditional methods fail spectacularly here. For instance, IW’s weights can explode when source clusters fail to cover target areas—reaching 6618% MAPE on Immune cells with a neural network (Table 3). NW also consistently underestimates risks, with errors ranging from 71.9–192% across datasets. KMM succeeds by explicitly matching the spatial structure of distributions through their kernel embeddings. For ecological or medical applications, this means that KMM can correct biases where traditional sampling underrepresented critical spatial regions, enabling more reliable risk estimates in underrepresented areas. For example, this study^37^ demonstrated that pneumonia screening models fail significantly when overrepresentation of certain demographics creates spurious correlations, mirroring our findings with IW’s MAPE> 6000% under spatial changes. KMM’s kernel-based matching avoids such pitfalls by explicitly aligning distributions without relying on biased density estimates, enabling reliable risk predictions even in underrepresented regions.

These findings underscore that distribution shifts in spatial data, whether due to sampling bias, environmental gradients, or inherent biological or spatial structure, can severely distort standard risk estimation, particularly for methods like IW that rely on direct density ratio estimation. The Classifier-based approach offers a more robust alternative to NW and IW, but can still be less accurate than KMM. The ability of KMM to correct for such shifts by matching distributions in a kernel feature space, without requiring explicit density estimation or labeled target data for error calculation, makes it particularly well-suited for spatial modeling in fields such as ecology, environmental science, and medical imaging, where model reliability under changing conditions is crucial.

## Conclusions

We addressed the challenge of risk estimation under spatial covariate shift by formulating it as a sample-reweighting problem. Our systematic evaluation reveals that KMM consistently outperforms traditional methods across both synthetic and real-world spatial datasets. While standard estimations become significantly biased under distribution shifts, KMM provides a reliable solution.

Our analysis shows that KMM overcomes the fundamental limitations of density ratio approaches, such as NW, IW and classifier-based methods. Unlike these estimations, KMM employs a direct distribution matching paradigm. By avoiding explicit density ratio estimation, it ensures stability even under complex structural shifts like Variance Scaling or in high-dimensional settings. For instance, where traditional density estimation fails, leading to IW errors exceeding 6000% in 4D immune cell data, KMM remains robust.

Quantitatively, KMM reduces estimation errors by 12.3% to 86.5% compared to alternative methods. It consistently outperforms classifier-based reweighting, highlighting that high discriminative accuracy alone is insufficient for proper distribution alignment. To assist in diagnosing these shifts, we integrated the LCF into our framework. Our results confirm that LCF is an effective, interpretable measure of spatial clustering that indicates the magnitude of a shift and signals when reweighting is necessary.

Our findings have immediate relevance for multiple domains. In ecological modeling, KMM can compensate for sampling biases in species distribution data. In biomedical applications, particularly spatial omics, it addresses significant variations in cell-type representation. Finally , this approach offers a robust solution for any field dealing with spatially heterogeneous data.

## Supplementary Information

Supplementary Information.

## Data Availability

For the data, preprocessing and modeling details to reproduce the calculations, we refer the reader to the repository of the project https://github.com/awesomeslayer/Importance-reweighting.
