# Supplementary material for: Kernel mean matching enhances risk estimation under spatial distribution shifts
Source: Sci Rep. 2026 Feb 2;16:6921. doi: 10.1038/s41598-026-36740-7 (PMC12917278; doi:10.1038/s41598-026-36740-7)
Supplement: Supplementary file 1 — Supplementary Information. [file 41598_2026_36740_MOESM1_ESM.pdf]

Supplementary material

Table S1. Overall Average ROC AUC scores for predictive models across datasets

Data Type	Model	2D	3D	4D	All Features
Species	Gradient Boosting	0.812	0.922	0.866	0.901
	Logistic Regression	0.756	0.771	0.722	0.771
	Random Forest	0.799	0.833	0.801	0.792
	Neural Network	0.805	0.850	0.820	0.830
Immune Cells	Gradient Boosting	0.749	0.762	0.718	0.770
	Logistic Regression	0.831	0.912	0.881	0.912
	Random Forest	0.783	0.759	0.722	0.899
	Neural Network	0.820	0.821	0.761	0.726

Table S2. Table of average ROC-AUC for trained Gradient Boosting classifiers for the classifier-based method across different data types and scenarios.

**Data Type / Scenario**	**2D**	**3D**	**4D**	**All Features**
Artificial Data Scenarios				
Domain Truncation: Cropped $\rightarrow$ Full	0.85	0.86	0.87	-
Domain Truncation: Full $\rightarrow$ Cropped	0.85	0.86	0.87	-
Mode Complexity: Expansion	0.84	0.85	0.86	-
Mode Complexity: Contraction	0.84	0.85	0.86	-
Correlation Shift: Diagonal $\rightarrow$ Non-Diag	0.83	0.84	0.85	-
Correlation Shift: Restoration	0.83	0.84	0.85	-
Variance Scaling: Extrapolation	0.84	0.85	0.87	-
Variance Scaling: Focusing	0.86	0.87	0.88	-
Support Mismatch: GMM $\rightarrow$ Uniform	0.84	0.85	0.87	-
Support Mismatch: Uniform $\rightarrow$ GMM	0.88	0.89	0.90	-
Real Data				
Immune Cells	0.85	0.86	0.86	0.88
Species	0.87	0.96	0.87	0.88

Table S3 Average true risk (R_{selected}) for sets of $K = 3$ models (values multiplied by 1000)

**Data Type**	**Dimensionality**	**NW**	**IW**	**KMM**	**Classifier**
Species	2D	2.658 ± 0.93	3.011 ± 1.14	**1.293 ± 0.58**	2.474 ± 0.87
	3D	5.211 ± 2.08	6.989 ± 2.80	**1.512 ± 0.68**	4.781 ± 1.82
	4D	6.634 ± 2.65	8.587 ± 3.43	**2.428 ± 1.09**	5.429 ± 2.17
	ALL	12.513 ± 4.38	13.591 ± 4.76	**7.541 ± 2.64**	10.911 ± 3.82
Immune cells	2D	2.851 ± 1.14	4.968 ± 2.18	**1.981 ± 0.89**	2.699 ± 1.05
	3D	4.887 ± 1.95	5.821 ± 2.33	**3.213 ± 1.45**	4.672 ± 1.82
	4D	5.518 ± 2.21	6.281 ± 2.51	**3.552 ± 1.60**	5.701 ± 2.28
	ALL	7.621 ± 3.05	10.832 ± 4.33	**4.711 ± 1.98**	6.913 ± 2.77

Table S4 Average true risk (R_{selected}) for sets of $K = 10$ models (values multiplied by 1000)

Data Type	Dimensionality	NW	IW	KMM	Classifier
Species	2D	2.131 ± 0.13	2.205 ± 0.14	**1.889 ± 0.09**	2.118 ± 0.12
	3D	3.755 ± 0.20	4.101 ± 0.24	**2.311 ± 0.10**	3.582 ± 0.19
	4D	4.951 ± 0.25	5.613 ± 0.29	**3.532 ± 0.16**	4.319 ± 0.21
	ALL	10.311 ± 0.41	10.789 ± 0.46	**8.921 ± 0.31**	9.588 ± 0.36
Immune cells	2D	2.321 ± 0.12	3.198 ± 0.19	**2.529 ± 0.10**	2.411 ± 0.11
	3D	4.012 ± 0.19	4.305 ± 0.21	**3.921 ± 0.15**	4.099 ± 0.18
	4D	4.698 ± 0.22	4.955 ± 0.24	**4.381 ± 0.17**	4.901 ± 0.20
	ALL	6.321 ± 0.29	7.844 ± 0.38	**5.989 ± 0.22**	6.205 ± 0.27

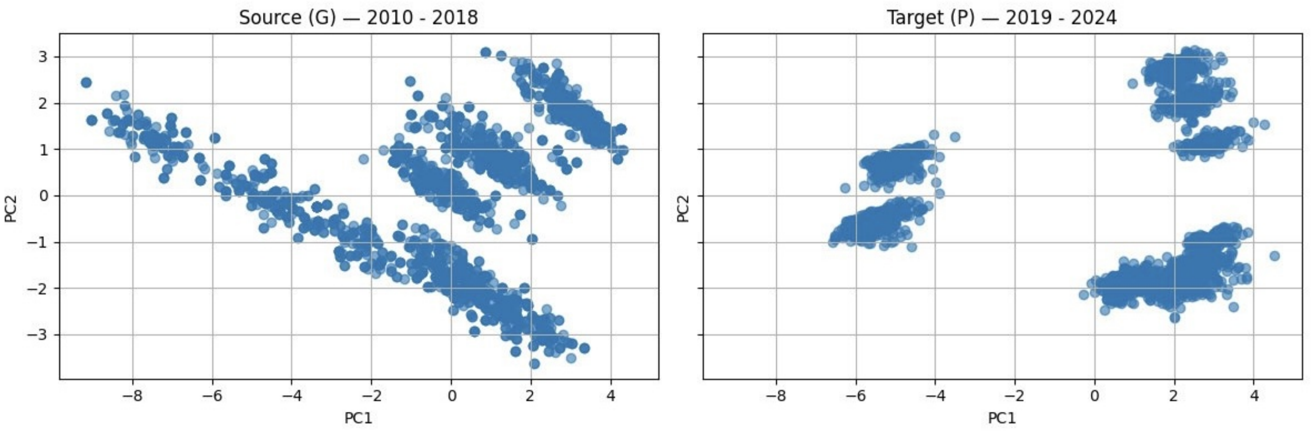

Figure S1. 2D PCA visualization of a representative species dataset pair. The source data (left) exhibits more distinct clustering compared to the less clustered target data (right).

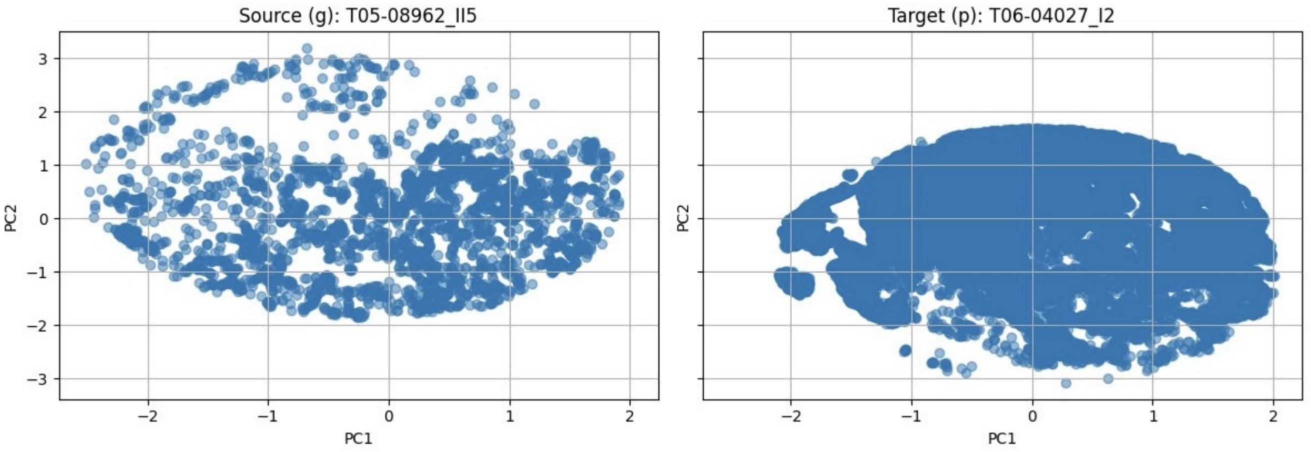

Figure S2. 2D PCA visualization of a representative cell dataset pair. Similar to the species data, the source data (left) shows clearer clustering than the target data (right).

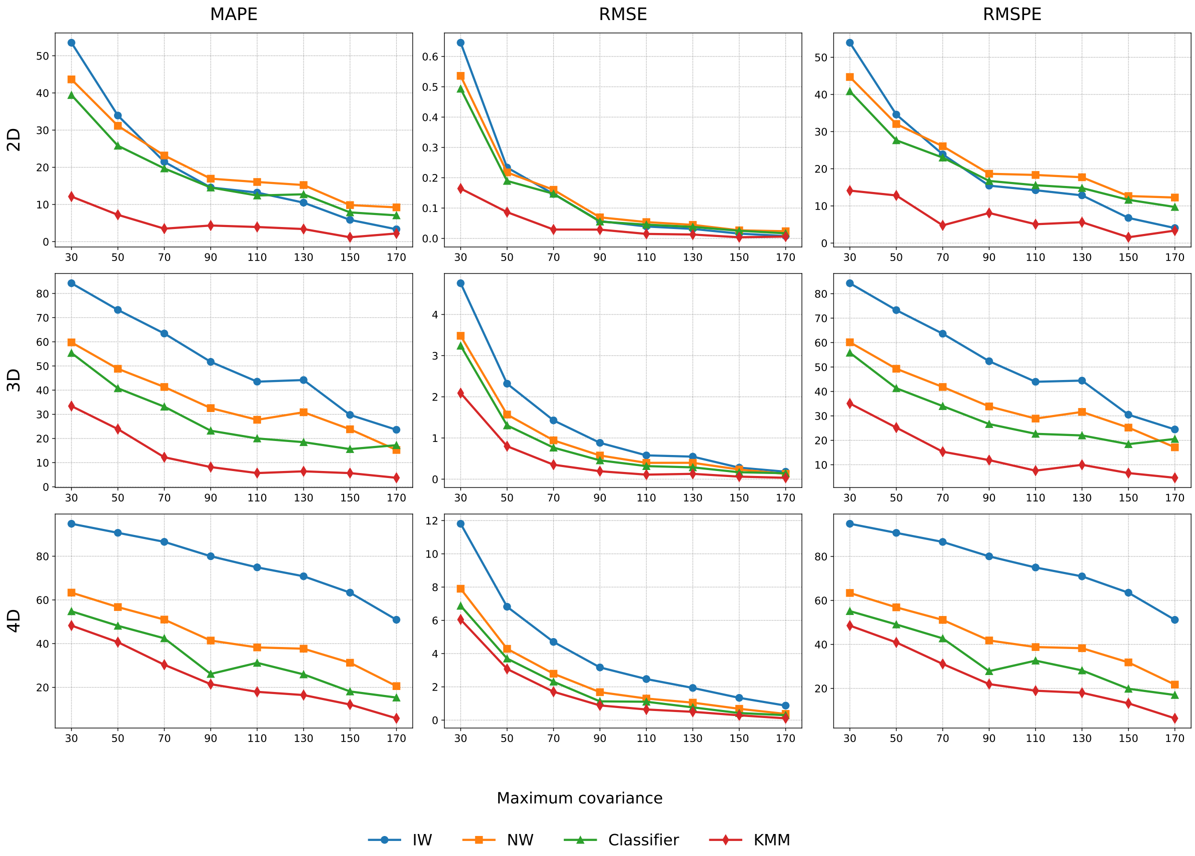

Figure S3. Performance metrics across varying maximum covariance values for the different Clustered GMM Source vs. Uniform Target scenario. Lower covariance indicates higher source clustering.

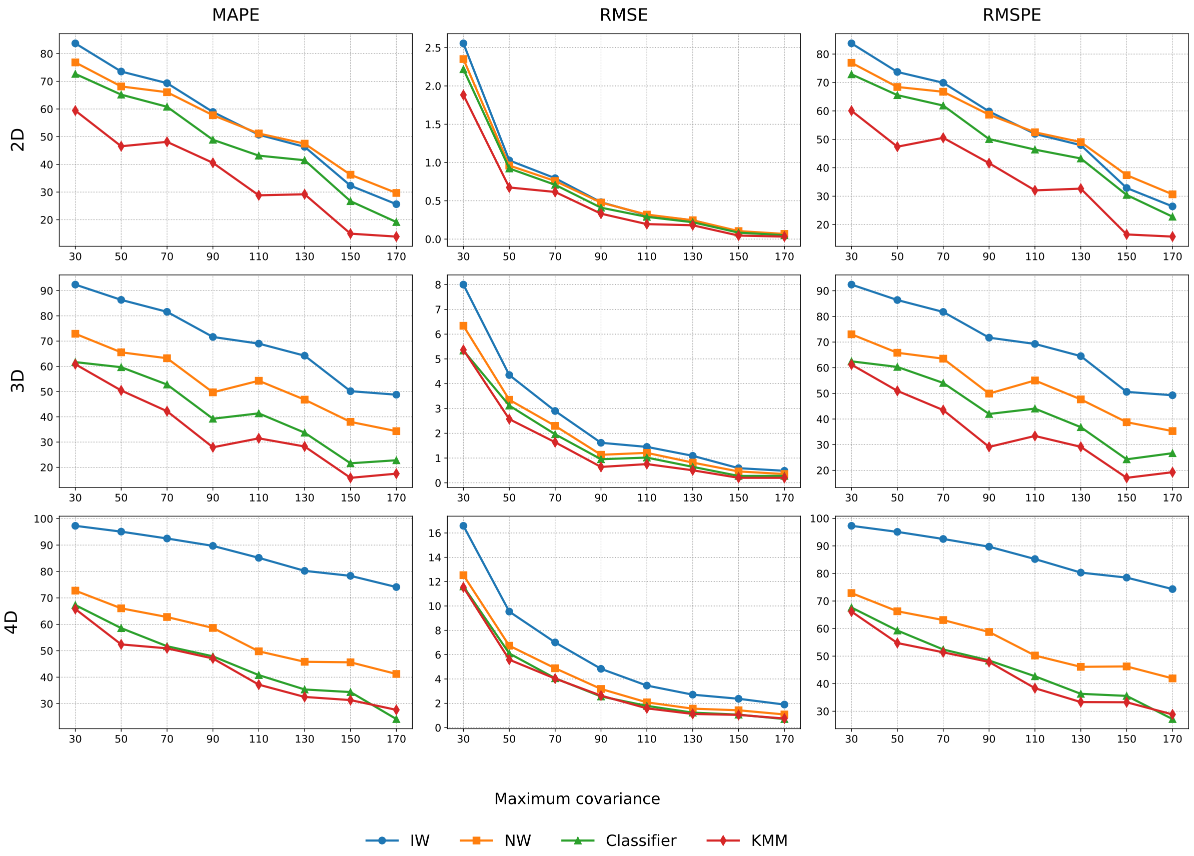

Figure S4. Performance metrics across varying maximum covariance values for the different Clustered GMM Source vs. Sparse GMM Target scenario. Lower covariance indicates higher source clustering.

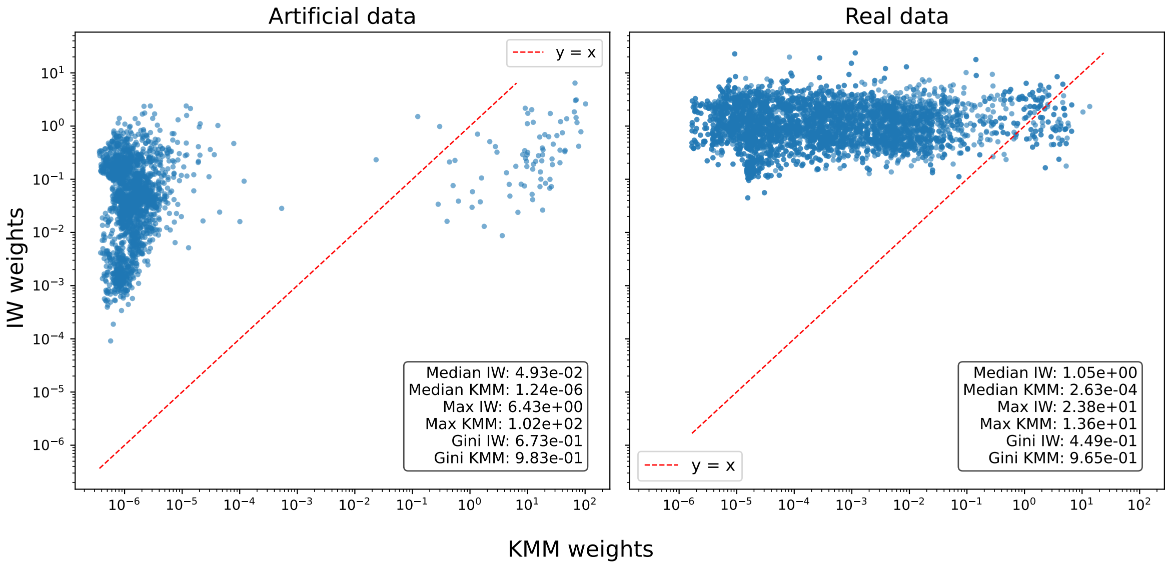

Figure S5. Comparison of individual sample weights produced by KMM (x-axis) and IW (y-axis) on representative artificial and real datasets. Each point corresponds to a single sample with coordinates (KMM_i, IW_i). The dashed line denotes y = x. Summary statistics (median, max, Gini) for each method are shown in the inset.

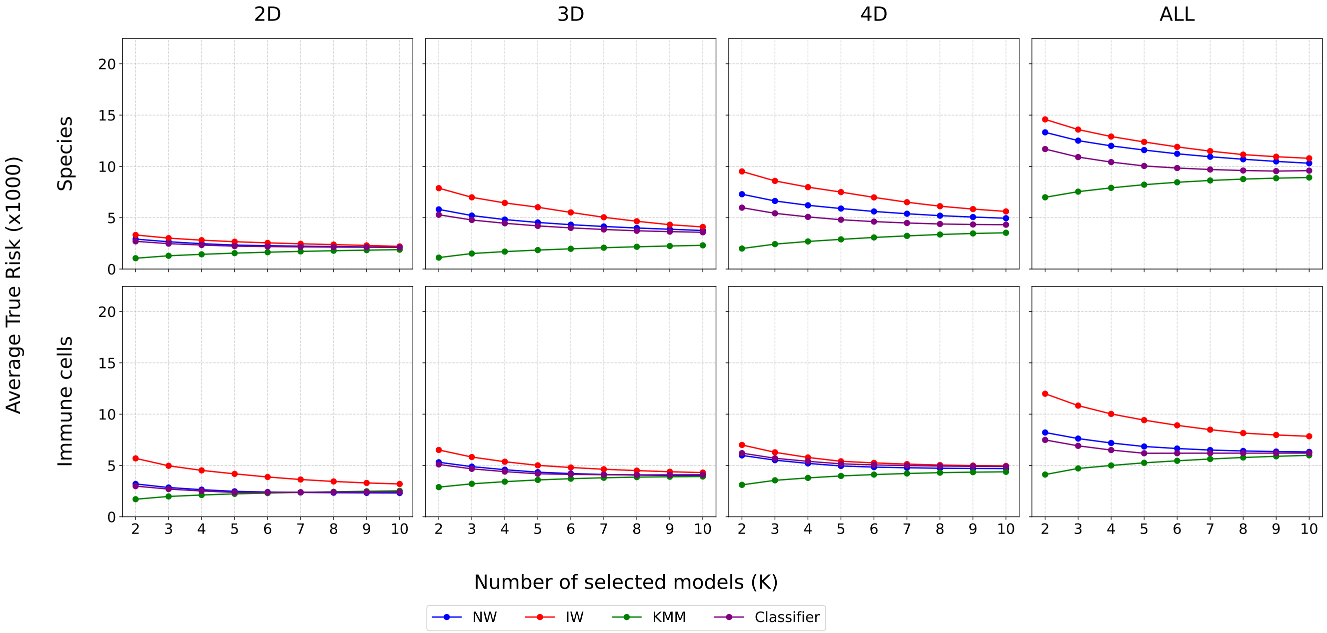

Figure S6 Average true risk (R_{selected}) as a function of the number of selected models (K) from 2 to 10. The plots illustrate the convergence of different model selection methods (NW, IW, KMM, Classifier)

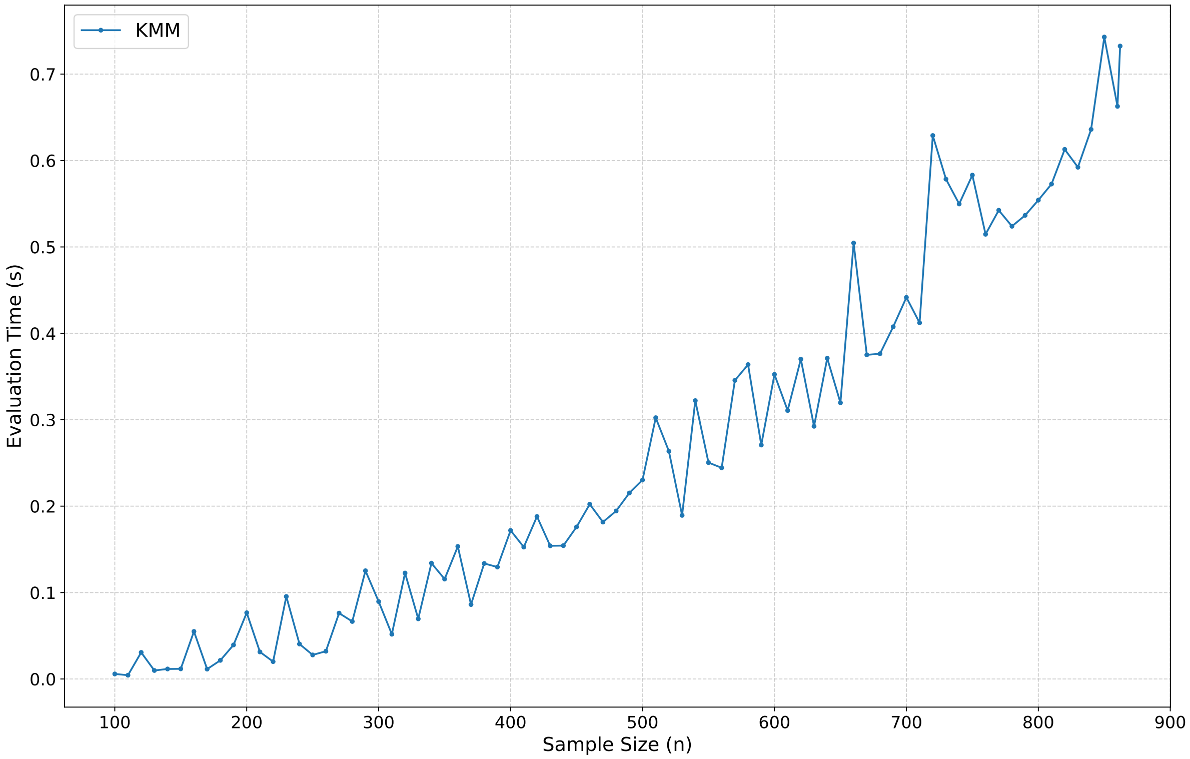

Figure S7. The execution time of the KMM method for the number of samples on Real data evaluation
